# Structures of the metallic and superconducting high pressure phases of solid CS_2_

**DOI:** 10.1038/srep10458

**Published:** 2015-05-18

**Authors:** Niloofar Zarifi, Hanyu Liu, John S. Tse

**Affiliations:** 1 Department of Physics and Engineering Physics, University of Saskatchewan, Saskatoon, Canada *S7N 5E2*

## Abstract

First principles structural prediction and molecular dynamics (MD) calculations have been performed to examine the structures responsible for the recently reported metallic and superconducting phases of highly compressed CS_2_. The low pressure experimental molecular crystal structure was found to be metastable and transformed into a disordered structure above 10 GPa. At 60 GPa, the predicted low energy structures show molecular CS_2_ is separated into C and S dominant regions. A crystalline structure with the *P*2_1_/*m* symmetry was found to be most stable from 60 to 120 GPa. The structure is formed from alternate layers of hexagonal C rings and S 2D-square-nets linked by C-S bonds. A non-crystalline structure with similar features structure is also predicted by MD calculations. Electron-phonon coupling calculations show this crystalline phase is superconductive. Contrary to the suggestions made from the experiments, no magnetism was found in all predicted low enthalpy high pressure structures. Moreover, the theoretical results do not support the proposal on the existence of hypervalent 6-coordinated carbon at 120 GPa.

## Introduction

CS_2_ is a transparent liquid under ambient conditions. It transformed into a molecular solid with the *Cmca* structure at 1 GPa. This phase is stable up to 9 GPa.^1^,^2^ Further compression resulted in a highly reflecting extended non-molecular solid above 40-50 GPa^3^. During this pressure range, the electrical resistance decreases continuously and an insulator-metal transition was found at ~50 GPa.^3^ A recent report further established that the metallic CS_2_ phase is a superconductor with a critical temperature (*T*_*c*_) of 6 K which remains almost constant from 60 GPa to 170 GPa.^4^ The latest study also revealed some surprisingly findings, for example, it was suggested magnetism existed in the normal state above 100 GPa and the structure is composed of 6-fold coordinated carbon atoms. There is little information on the structure of the high pressure phases. Above 9 GPa, x-ray diffraction^3^ measurements show intensity of the Bragg diffraction peaks weakened and the line-widths broadened indicating a gradual transformation into an amorphous form. Up to 120 GPa, no features characteristic of elemental carbon and sulfur were observed. The objectives of this study are (i) to explore candidate structures responsible for the superconducting behaviour; (ii) to examine possible magnetic electronic states and (iii) to investigate the existence of unprecedented hypervalent six coordinated carbon atoms in the structures. For these purposes, first-principles MD and structure prediction calculations using evolution algorithm (GA) and particle-swarm optimization (PSO) were performed on stoichiometric 1:2 carbon sulfur crystalline phases (CS_2_). Both methods predicted structures with distinct carbon and sulphur domains are favoured at high pressure. Moreover, a crystalline structure with a 2-D sulphur network and diamond like carbon chains was found to be most stable between 60 and 120 GPa. This structure is metallic and superconductive. Within the experimental pressure range, no evidence of six coordinated carbon or magnetism was found in any of the predicted structures.

## Results

**Figure 1:**
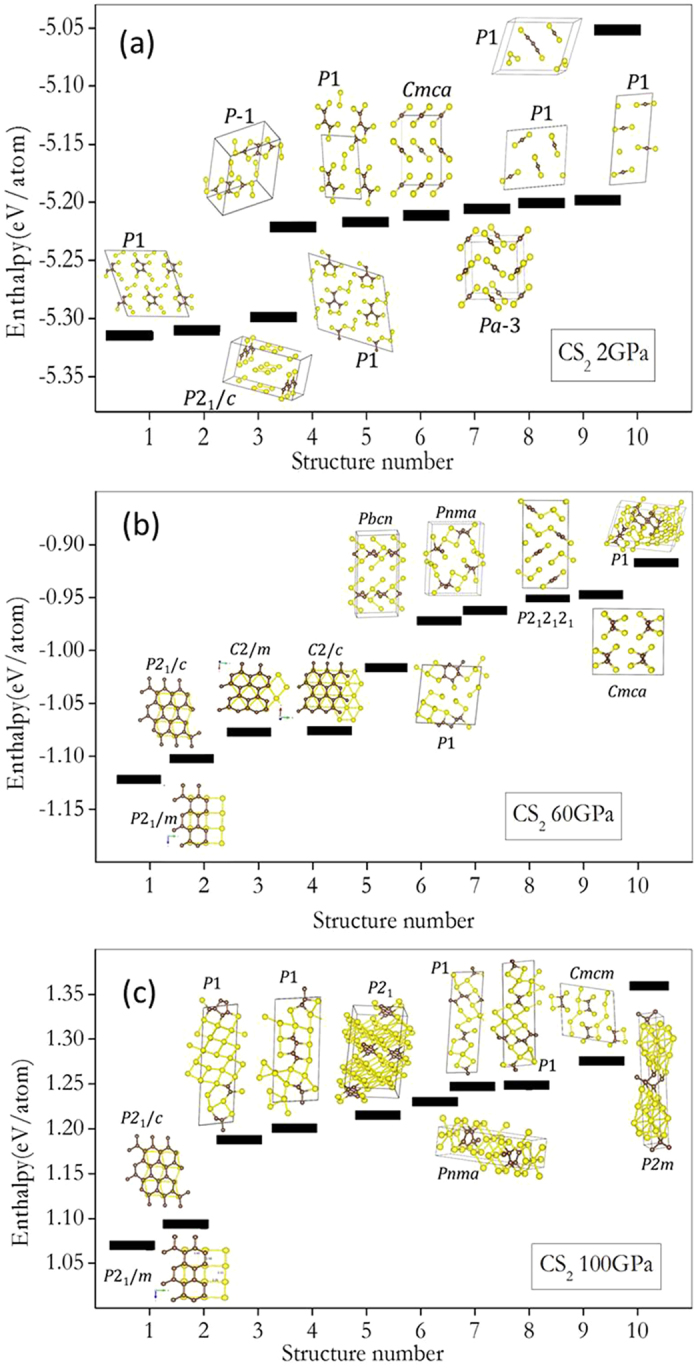
Predicted enthalpy structures for solid CS_2_. 10 lowest predicted enthalpy structures for solid CS_2_ at (a) 20 GPa, (b) 60 GPa and (c) 100 GPa).

First we tested the structural prediction methods on the known molecular CS_2_ crystal structure at 2 GPa. The observed *Cmca* structure was found readily by the PSO search. GA calculations also produced several crystalline structures but with slightly higher energies and the CS_2_ molecules packings were different to the *Cmca* (Fig. 1a). Interestingly, the *Cmca* structure is metastable and is about 0.1 eV/atom higher than the global minimum. A number of lower enthalpy structures characterized by the existence of molecular fragments composed of C-C, S-S, C-S bonds and C-S cyclic rings (Fig. 1a) were found. At first glance, the result seems to be erroneous. Simple empirical bond energy consideration however shows structures with C-C chains and C and S ring units are energetically more favourable than those composed solely of molecular CS_2_. Moreover, a large number of predicted structures have low space group symmetries (i.e.P1) indicting that these structures are probably disordered. Experimentally, crystalline CS_2_ was obtained from the condensation of CS_2_ molecules. A large energy barrier is needed to break the C=S bonds into molecular fragments of the extended structure and that explains the metastability of the molecular crystal structure. The preference towards the formation of connected extended structures has also been found in a recent theoretical study on solid carbon monoxide (CO). The molecular structure is only found to be stable at ambient pressure. At higher pressures, polymeric C-O structures are preferred since the C-O triple bond is not energetically competitive with the formation of C-O chains.^5^ The different low pressure crystalline structures predicted by PSO and GA methods prompted us to adopted an alternative protocol for structural search in the ensuing calculations. First, structural searches were performed using the PSO and GA methods independently in the normal manner. If the two methods produced different energetically most stable structures, these structures were then introduced into both populations and the searches were repeated. In this way one can increase the probability that the predict structure is the global minimum.

**Figure 2:**
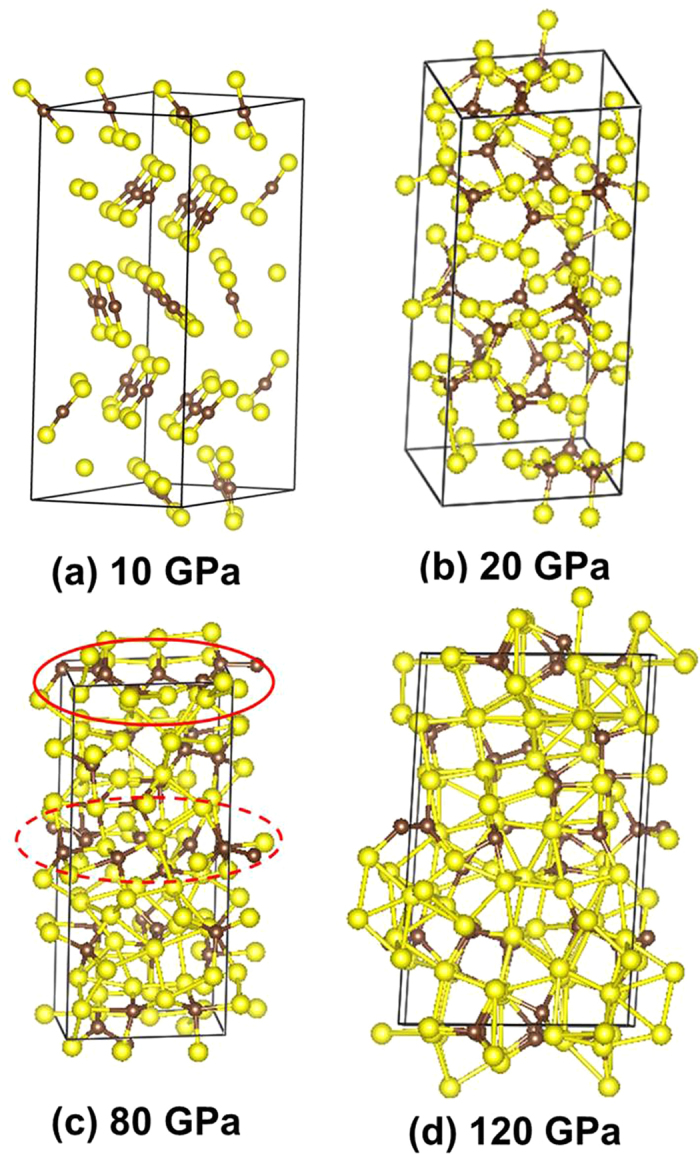
Snapshot of molecular dynamics calculations. (a) 10 GPa, (b) 20 GPa, (c) 80 GPa and (d) 120 GPa. The red circle highlights the formation of C-C “clusters” at 80 GPa. The occurrence of S square nets is clearly seen at 120 GPa.

To search for possible structures of the high-pressure metallic phase, we first compressed the molecular CS_2_ structure with a constant-pressure–constant pressure (*NPT*) molecular dynamics (MD) calculation at 300K. The crystalline structure was found to transform into a disordered 3D solid connected by C-C, C-S and S-S bonds (Fig. 2b) at 20 GPa. Further compression to 80 GPa reveals the occurrence of segregated C and S regions in the solid (Fig. 2c). At 120 GPa, planar square networks formed by S atoms in the disordered structure are almost fully developed (Fig. 2d). To define the underlying crystalline structure, structural prediction calculations following the procedure described above were performed at 60 GPa. A crystalline structure with the *P*2_1_/*m* space group was found to be most stable (Fig. 1b). This structure becomes more stable than the crystalline molecular *Cmca* phase at pressure higher than 10 GPa. It is constructed from a C-C layer sandwiched between two S layers linked in the third dimension by C-S bonds. In a way, this structure feature is similar to the observation made in the MD calculation. It should be noted that structures found in a MD calculations are often the ones with low kinetic barriers from the initial structure. So it is not surprising that the results of MD calculations are different from that of GA and PSO predictions. In the evolution structure searches, both GA and PSO are designed to find the lowest enthalpy structure. In this case, MD calculations reveal a metastable structure trapped in a local minimum that did not fully transform to the covalent carbon-rich structure, possibly due to the limited size of the model and the lack of atom thermal mobility. However, both MD and evolution structure searches suggest that CS_2_ will adopt a sulphur layer structure and, at high pressure, molecular CS_2_ separated into solid sulphur and carbon rich compounds. The 2D S layers form almost planar 2D networks as in hexagonal diamond (Fig. 1). The C-C layer is consisted of hexagonal rings in a chair conformation. Within an energy difference of 0.1 eV/atom, several structures with slightly higher enthalpies were found. Remarkably, a *P*2_1_/*c* structure with a very similar bonding pattern as the *P*2_1_/*m* was found. The major differences between the two structures are that in the latter, the squares in 2D S layer is slightly distorted to parallelograms and the C atoms forming the hexagonal ring are arranged in the boat conformation as in cubic diamond. However, the *P*2_1_/*c* structure is found to be dynamically unstable (Fig. 1S). In order to accommodate chemical bonding with the C layer arranged in the chair form in the *P*2_1_/*c* structure the square net in S layer is distorted. In comparison, the square-nets in the *P*2_1_/*m* structure fit well to bond with C atoms in the chair conformation. The fact that small difference in the 2D packing of S layers can affect the energetic of the crystals illustrates that C-S bonding is an important to the stability of the high-pressure phases. Predicted structures within a small energy interval often clustered into groups and with similar local bonding patterns. For example, at 60 GPa, two additional structures within <0.05 eV/atom (*C*2/*m* and *C*2/c) of *P*2_1_/*m* structure with very similar packing patterns were found. The major difference of these structures from *P*2_1_/*m* is increasing closed pack from squares to rhombus (*C*2/*m*) and hexagons (*C*2/*c*) in the S layers. As even higher energy, the chemical bonding of the solids changed to a group of structures with disconnected mixed C-S regions and finally molecular fragments with C-C bonds start to emerge. At this pressure, CS_2_ molecules decomposed into segregated C and S regions.

**Figure 3:**
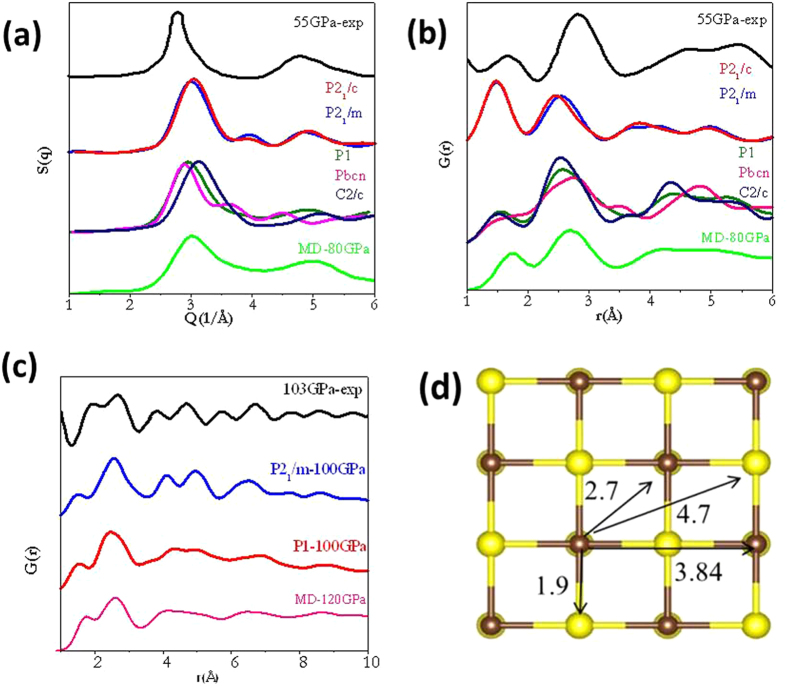
Radial distributional function of solid CS_2_. (a), (b) and (c) the radial distributional function of solid CS_2_ compared with experimental data at 55 GPa and 103 GPa. (d) Six coordinated C structure of CS_2_ proposed in ref. 4.

There is limited experimental information on the structure of the high-pressure metallic phase of CS_2_. X-ray powder diffraction experiments performed at 55 GPa shows two broad peaks centered at 2.8 and 4.8 Å^−1^ is typical of a disordered solid^3^. In the previous study, following the high pressure transformation sequence observed in the analogous solid CO_2_, it was proposed that the disordered phase has a distorted tridymite *P*2_1_2_1_2_1_ structure.^6^ This structure was not found in the search. Separate calculations show it has a substantially higher enthalpy of 0.2 eV /atom than the *P*2_1_/*m* structure. To compare with the experiment, *S*(*q*) for the crystalline *P*2_1_/*m* (broadened by a linewidth of 0.5 Å^−1^ to mimic potential disorder) and several low enthalpy structures were calculated (Fig. 3a). Similar to the experiment, all the calculated diffraction patterns show two bands at ~2.8 and 4.8 Å^−1^. The *P*2_1_/*m* structure shows an additional weak feature at ~4 Å^−1^. The calculated pair distribution functions (PDF), *G*(*r*), are in agreement with the experimental assignment (Fig. 3b). Previously, the first peak in the radial distribution function (RDF) was assigned to nearest C-S distance at ~1.7 Å and the second peak to neighboring S-S distances at around 2.77 Å.^3^ These two features may be related to the calculated peaks at 1.5 and 2.6 Å. Inspection of the lowest energy structures attributes the first peak in the PDF to C-C bonds where the S-S distances are between 2.13-2.89 Å and the second nearest neighbour C-C and C-S bonds distributed between 2.33-2.81 and 2.56-2.95 Å respectively. The *S*(*q*) and *G*(*r*) for the MD structure and the structural assignments (Fig. 3) are also in agreement with the predicted structures and the experiment. Unfortunately, the limited information from experiment precludes an unambiguous determination of the structure.

## Discussion

To investigate the suggestion of hypervalent carbon atom in the structure above 100 GPa, structural search were performed following a similar procedure described above. It is found that the *P*2_1_/*m* crystalline structure remained to be the most stable. This is followed by the *P*2_1_/*c* structure. In the next group of structures with much higher enthalpy (<0.12 eV/atom), instead of separate C and S layers, they consist of C clusters embedded in the 2D plane composed of S atoms (Fig. 1c). Theoretical pair distribution function *G*(*r*) of several low enthalpy structures were computed and compare with results obtained from experimental diffraction patterns at 103 GPa (Fig. 3c). In the experimental paper, the peaks at 1.9 and 2.7 Å were assigned to first nearest neighbour C-C, C-S distances and the second nearest neighbour C-C at 3.84 Å and C-S at 4.7Å. Based on the assignments an octahedral local configuration with six-coordinated C atoms (Fig. 3d) was hypothesized. The calculated *G*(*r*) reproduced all the main features in the observed distribution function. Once again, the structure obtained from the MD calculations support these assignments. Analysis shows the first observed peak can be attributed to the C-C distances. However, the second broad peak contains contributions from both second nearest-neighbours of C-S and C-C and the first nearest-neighbour S-S distances. Although the second nearest neighbour C-C is located at 3.84 Å in the crystalline *P*2_1_*/m* structure, the dominant peak in the experimental *G*(*r*) also contains overlapping C-C, C-S and S-S bond distances. The peak at 4.7Å is assigned to the next nearest C-S separation. We have also examined predicted structures with much higher enthalpy. All features in the *G*(*r*) derived from the experimental diffraction pattern can be explained adequately with a structure consisted of solely tetrahedral coordinated C atoms. No evidence on the existence of six coordinated carbon atoms was found. Moreover, the proposed structural pattern constructed from alternate C and S atoms at the corners (Fig. 3) of a square net were not found in any of the predicted structures.

The *G*(*r*) based on the diffraction pattern provides information on atom arrangement in the short and intermediate order. Undoubtedly, the experimental high pressure structure is non-crystalline, however, in view of the gross agreement between the experimental and calculated *G*(*r*) the predicted crystalline *P*2_1_/*m* and several energetically competitive structures and with the disordered structure obtained from MD calculations, it is probable that the observed structure is composed of segregated C-C and S-S regions linked by C-S bonds with the latter forming 2D square-nets or even micro-crystalline domains of the *P*2_1_/*m* phase. Apart from the structural similarities, the calculated electronic density of states of both the crystalline *P*2_1_/*m* and the disordered MD structure shows the electronic density of states (DOS) near the Fermi level is also dominated by the S valence 3*p* and 3*d* orbitals (Fig. 3 and S5) (*vide supra*). Since electron phonon coupling is determined by electronic states within a thin shell of the Fermi surface, it is not unreasonable to explore the origin of the superconducting behaviour in the experimental disordered phase using the crystalline *P*2_1_/*m* structure as a model.

**Figure 4:**
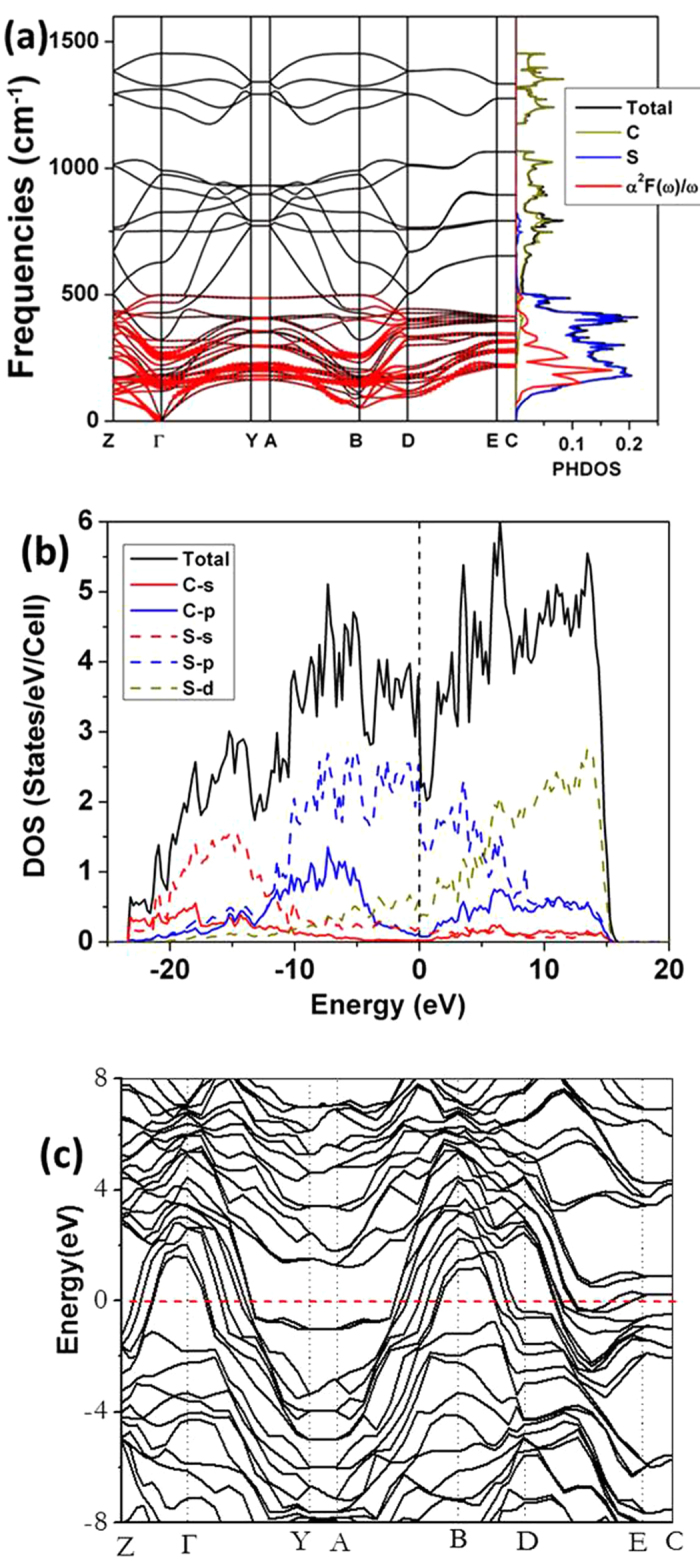
Phonon and electronic band structreus of CS_2_. Calculated (a) phonon band structure and Eliashberg spectral function (α^2^*F*(ω)). (b) and (c) electronic density of states and electronic band structure for the *P*2_1_/*m* structure at 60 GPa. Red solid circles in (a) show the magnitude of electron-phonon coupling with the radius proportional to the strength.

Density functional perturbation theory and frozen phonon calculations were performed to establish the stability of the *P*2_1_/*m* structure (Figs. 4a and 1S, respectively). At 60 GPa, no imaginary frequency is found with both methods indicating that the structure is dynamically stable. Soft phonon modes reminiscence of Kohn-anomalies are found at the B and D symmetry points (Fig. 4a). The projected vibrational density of states onto C and S atoms for the *P*2_1_/*m* phase was also presented. As expected the low frequency modes are dominated by S-S vibrations due to the heavier atomic mass. The DOS of the *P*2_1_*/m* structure depicted in Fig. 4b shows that it is metallic. The DOS near the Fermi energy is dominated by low-lying S-3*p* states. This is a consequence of the S regions in the structure, a distinctive feature shared by all the predicted low energy structures. For this purpose, the electron-phonon coupling parameter (λ) and the logarithmic average phonon frequency (ω_ln_) at 60 GPa were calculated using the linear response theory. The individual interatomic force constant matrix and electron phonon coupling matrix were calculated employing the linear response method at a 1×3×2 ***q*** -point mesh with a 4×12×8 ***k***–point mesh for the first Brillouin zone integrations, and the plane wave cutoff was chosen as 60 Ry. At 60 GPa, the calculated coupling parameter λ is 1.04 with an average phonon frequency ω_ln_ of 341 K. Using the strong coupling Allen-Dynes equation, an extension of the McMillan theory,^7^ and nominal Coulomb pseudopotential parameter (μ*) with values of 0.1, 0.12 and 0.13, the estimated superconducting critical temperature *T*_*c*_ are 25 K, 22.8 K and 19.5 K, respectively are slightly higher than the observed 5.6 K. The origin of the superconductivity is revealed from the calculated Eliashberg spectral function. α^2^*F*(ω)/ω.^8^ As shown in Fig. 4a, nearly 100% of the electron phonon coupling is contributed by S-S vibrations in the frequency region from 0–500 cm^−1^. The strong interactions led to a pronounced peak in the spectral function at 200 cm^−1^. In addition, we have computed the phonon line widths and nesting functions (Fig. 2S). In the square planar S-S layers strong nesting are found approximately midway from Γ → Y, D → E and at the B symmetry point (Fig. 2S). The superconductivity is the consequence of the S-S bands that dominate the Fermi level. Further electronic and superconductivity calculations on the *P*2_1_/*m* structure were also performed at 80 and 100 GPa. The electron and phonon band structures are very similar to that at 60 GPa (Fig. 4 and Figs. S3 and S4). It is remarkable that using a μ* of 0.12, the predicted *T*_c_ at both pressures are 13 K. The result in accord with the almost constant *T*_c_ of 6 K reported from 60-172 GPa.^4^ In passing, we have also performed spin unrestricted calculations on several low enthalpy structures and no magnetic state was observed. However, since the electronic calculations were performed at 0 K, the results do not preclude possible magnetism at finite temperature.

Structural search indicates that molecular CS_2_ will decompose and segregate into C and S regions in the solid at high pressure. The carbon atoms tend to form fused hexagonal rings either in the boat or chair conformation, akin to the hexagonal and cubic diamonds, respectively. On the other hand, the sulfur atoms adopt a planar closed pack arrangement forming 2D square or hexagonal networks. Intuitively, it is logical to expect it is energetic favorable to form regions with diamond-like and sulfur-like sub-structures at high pressure. A crystalline structure with the *P*2_1_/*m* space group was found to be most stable from 60-120 GPa. This structure is metallic and superconductive with a *T*_*c*_ of 20-13 K from 60-120GPa. The superconductivity is mainly the result of electron-phonon coupling in the S layers. The measured diffraction pattern (*S*(*q*)) and the derived pair distribution functions (*G*(r)) at 55 GPa and 103 GPa can be reproduced from predicted low enthalpy structures with 2D layers. The theoretical results show the low enthalpy structure is non-magnetic and no evidence of 6-coordinated C above 100 GPa was found.

Finally, we wish to emphasize that the present study was focused on C:S=1:2 stoichiometry. Although there is no direct experiment evidence, we cannot rule out the possible formation of C_x_S(X>1) species under high pressure. In view of the metallic and superconductive characters of the high pressure phases, if these C-rich species were ever formed, they must consisted of polymeric *sp*^2^ C=C. Obviously, further studies on variable C/S composition are needed in order to fully understand the intricate structure, superconductivity and magnetism of CS_2_ compounds at high pressure.

## Methods

All electronic calculations were performed using the Vienna *ab initio* simulation package (VASP),^9^,^10^,^11^ a plane wave code employing the projected-augmented wave (PAW) potentials^12^ based on the density functional theory with the Perdew-Burke-Ernzerhof (PBE)^13^ parameterization of the generalized gradient approximation (GGA). During the structural search, Monkhorst-Pack *k*-point grids^14^ were generated at a predefined mesh by scaling the reciprocal lattice vectors of each individual structure and increased in steps as the electronic structure geometry optimization calculation approach convergence. Structural searches were performed with the particle swarm optimization method implemented in the CALYPSO suite^15^ and genetic algorithm method with our ASAP code.^16^ Populations consisted of 40-50 trial structures on model systems up to eight formula units of CS_2_ (24 atoms) was used. A search was terminated after a minimum energy structure was found and no new lower enthalpy structure appeared for at least 20 subsequent generations. Usually, 50 to 60 generations were needed to achieve convergence.

## Conclusion

It is pertinent to comment on the structural similarity in the superconductivity^17^ phase-V of elemental sulphur^18^ with the disordered CS_2_. Under compression, S undergoes a serious of structural transformations. Between 83-253 GPa, a metallic state with superconductivity in an incommensurate structure was observed. A feature in common to the predicted disorder high pressure structure of CS_2_ presented here and S-V is the existence of closed pack S atom layers. In the latter case, the S atoms are arranged in a hexagonal closet pack but cubic pack in the latter. It is evident that delocalization of electrons in planar S-layers is the essential ingredient for the superconducting behaviour. Recently, very high *T*_c_ (*ca*. 190 K) has been found in hydrogen sulphide (H_2_S) compressed to 200 GPa. 19 It was suggested that the high-pressure phase is composed of decomposed H_2_S. From the results obtained here, we speculate that similar S-layers are formed and strong electron-phonon coupling in these layers and with the hydrogen atoms may be reason for the very high *T*_*c*_.

## Author Contributions

J.T. conceived the idea. N.Z. and H.L. performed the simulations. J.T., N.Z. and H.L. wrote the manuscript.

## Additional Information

**How to cite this article**: Zarifi, N. *et al.* Structures of the metallic and superconducting high pressure phases of solid CS_2_. *Sci. Rep.* **5**, 10458; doi: 10.1038/srep10458 (2015).

## Supplementary Material

Supplementary Information
