# Supplementary material for: Structures of the metallic and superconducting high pressure phases of solid CS2
Source: Sci Rep. 2015 May 18;5:10458. doi: 10.1038/srep10458 (PMC4434837; doi:10.1038/srep10458)
Supplement: Supplementary Information [file srep10458-s1.doc]

**Supplementary Material**

Structures of the metallic and superconducting high pressure phases of solid CS_2_

Niloofar Zarifi^1^, Hanyu Liu^1,*^ and John S. Tse^1,*^

^1^Department of Physics and Engineering Physics

University of Saskatchewan

Saskatoon, Canada S7N 5E2


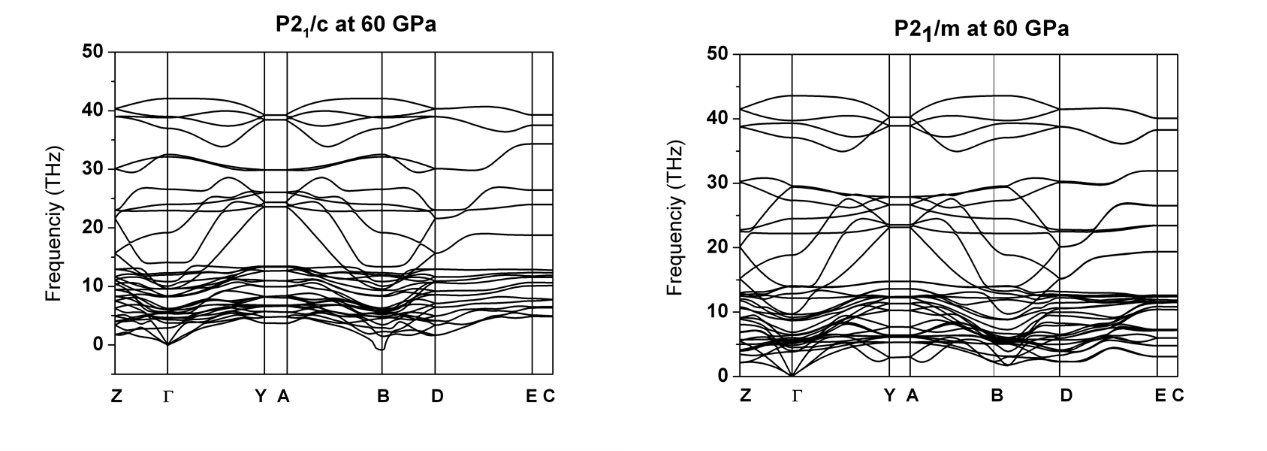


**Fig. S1** Calculated phonon structures of *P*2_1_/*c* structure and *P*2_1_/*m* structures.


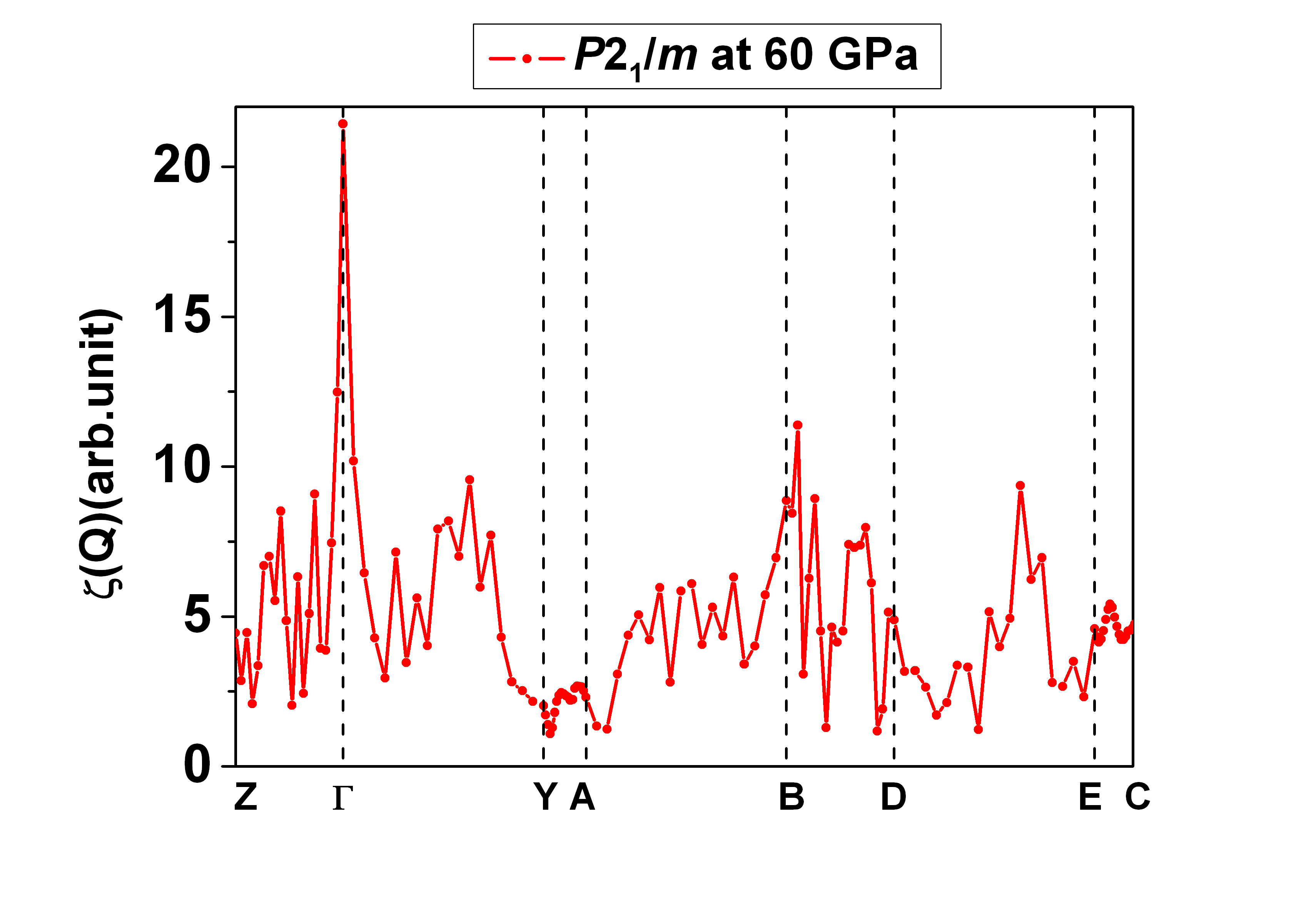


**Fig. S2** Nesting function of *P*2_1_/*m* structure at 60 GPa.


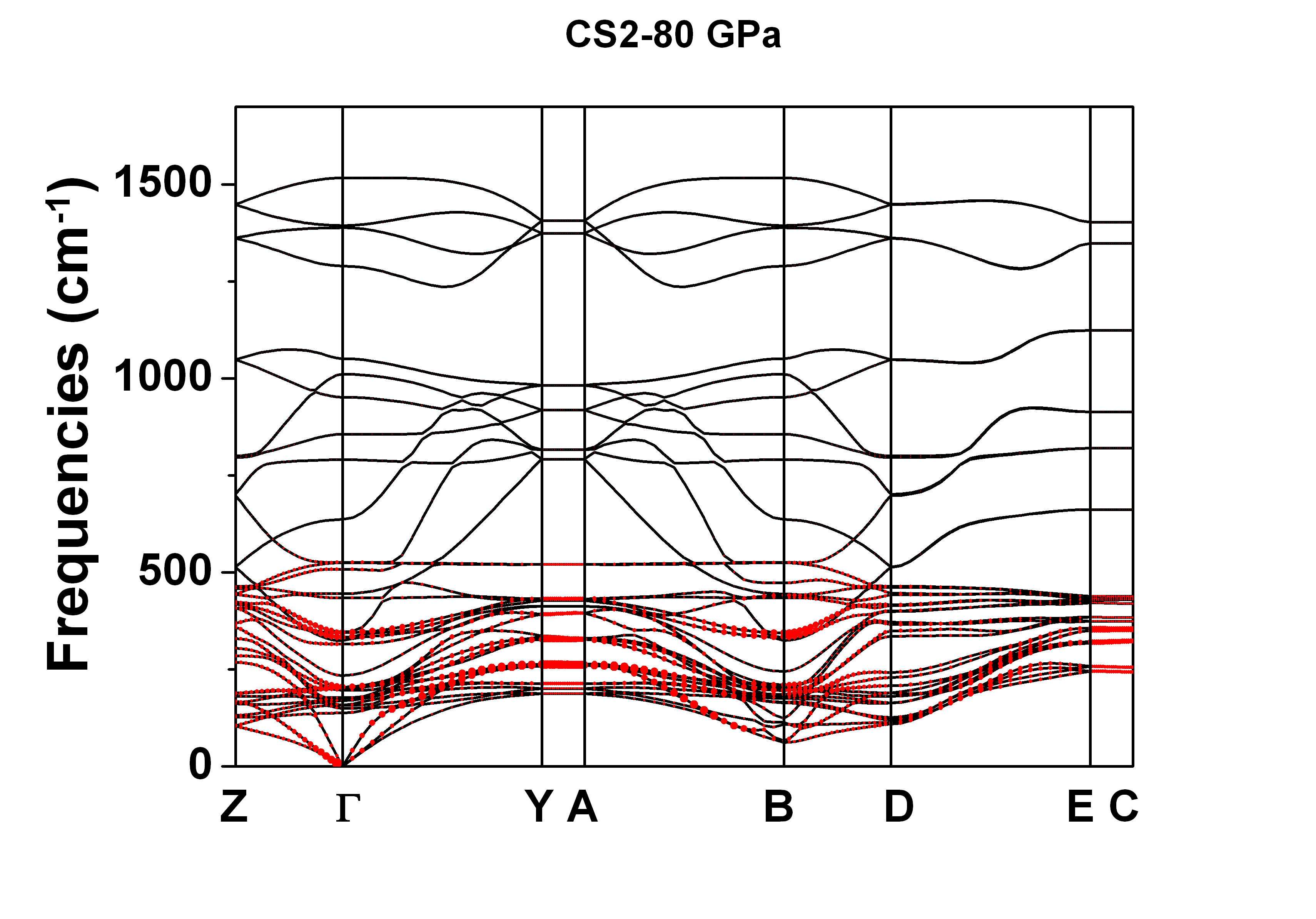


**Fig. S3** The phonon structure and electronic structure of *P*2_1_/*m* structure at 80 GPa.


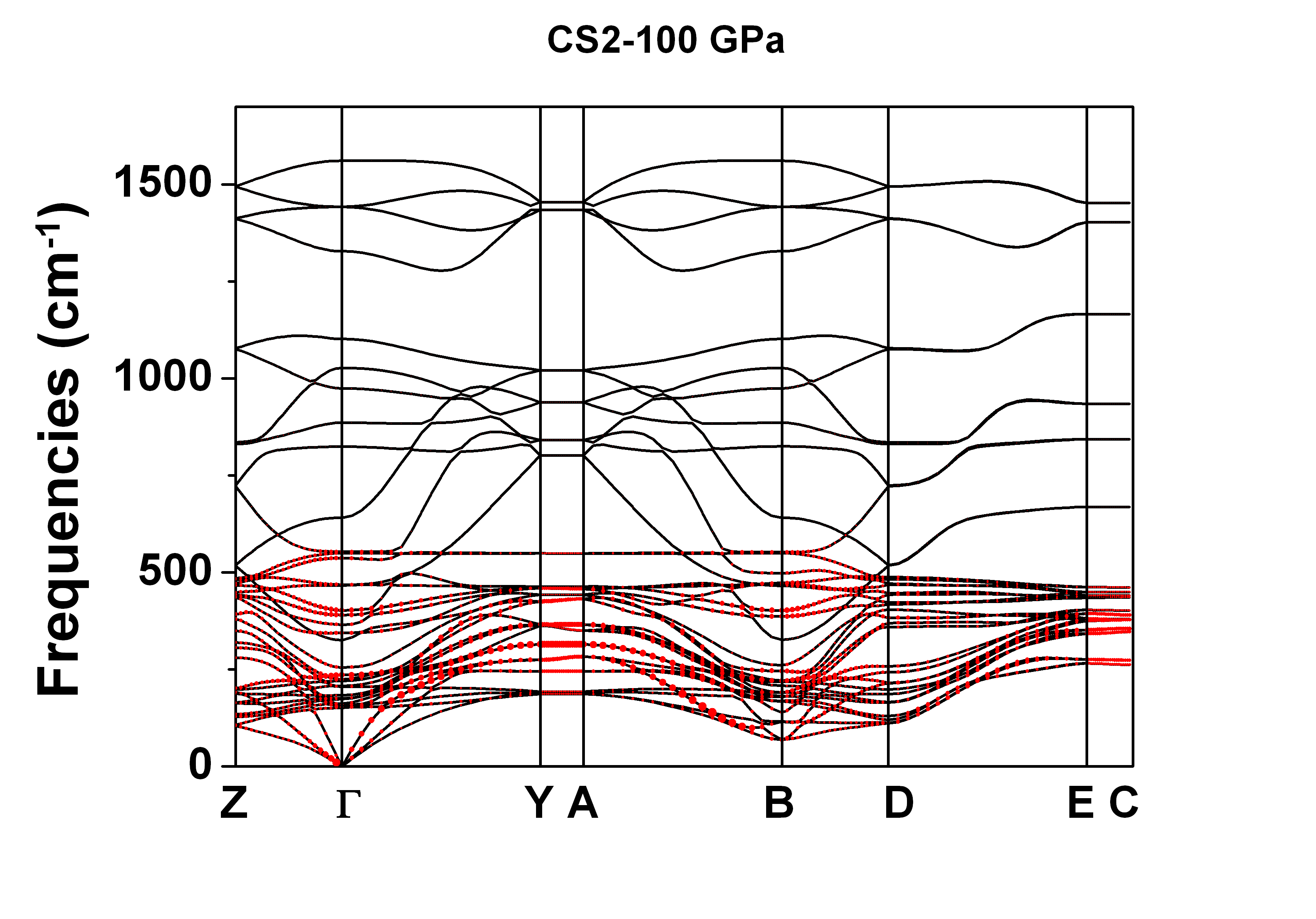


**Fig. S4** The phonon structure and electronic structure of *P*2_1_/*m* structure at 100 GPa.


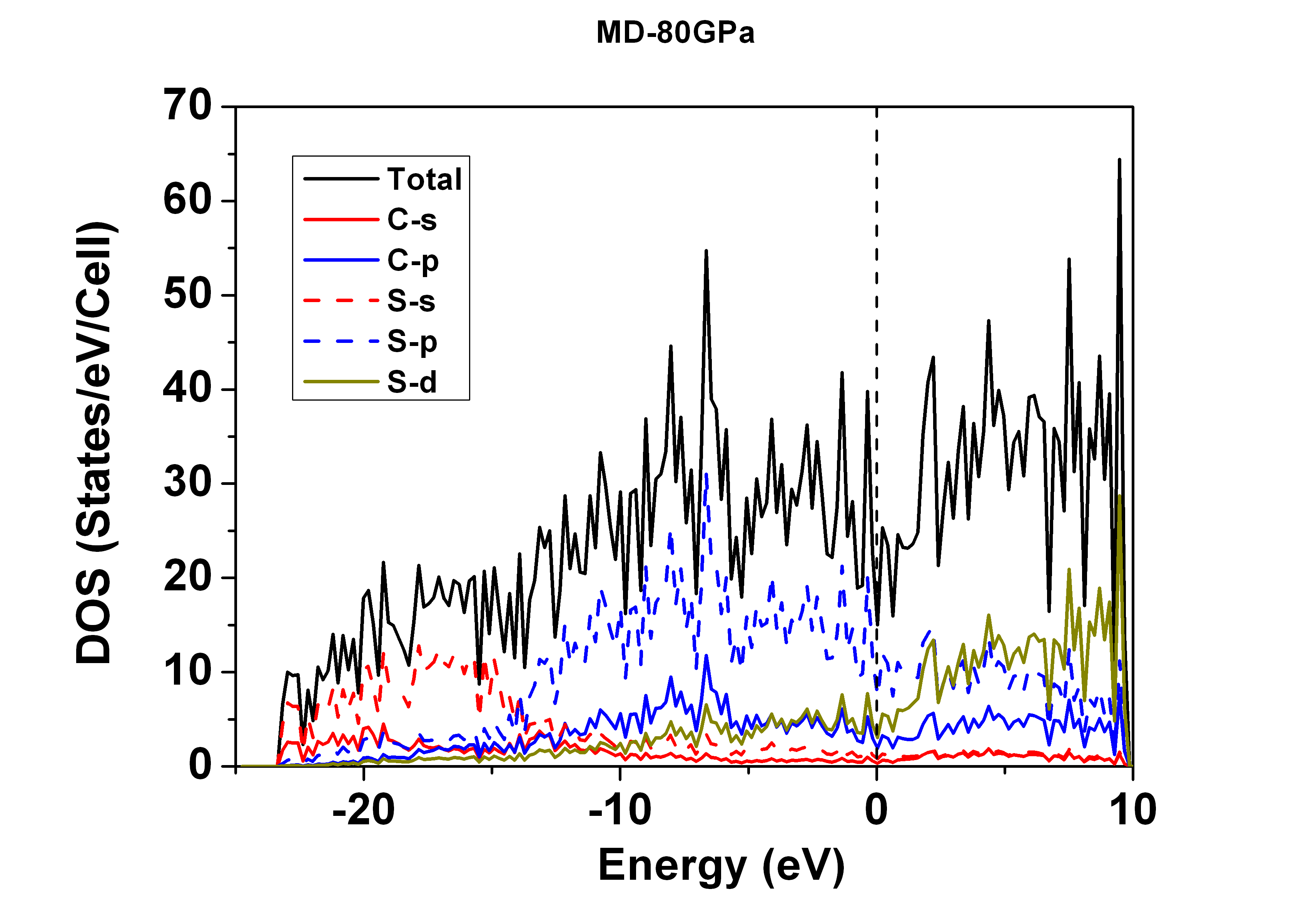


**Fig. S5** The calculated density of states of CS_2_ structrure by MD simulation at 80 GPa.


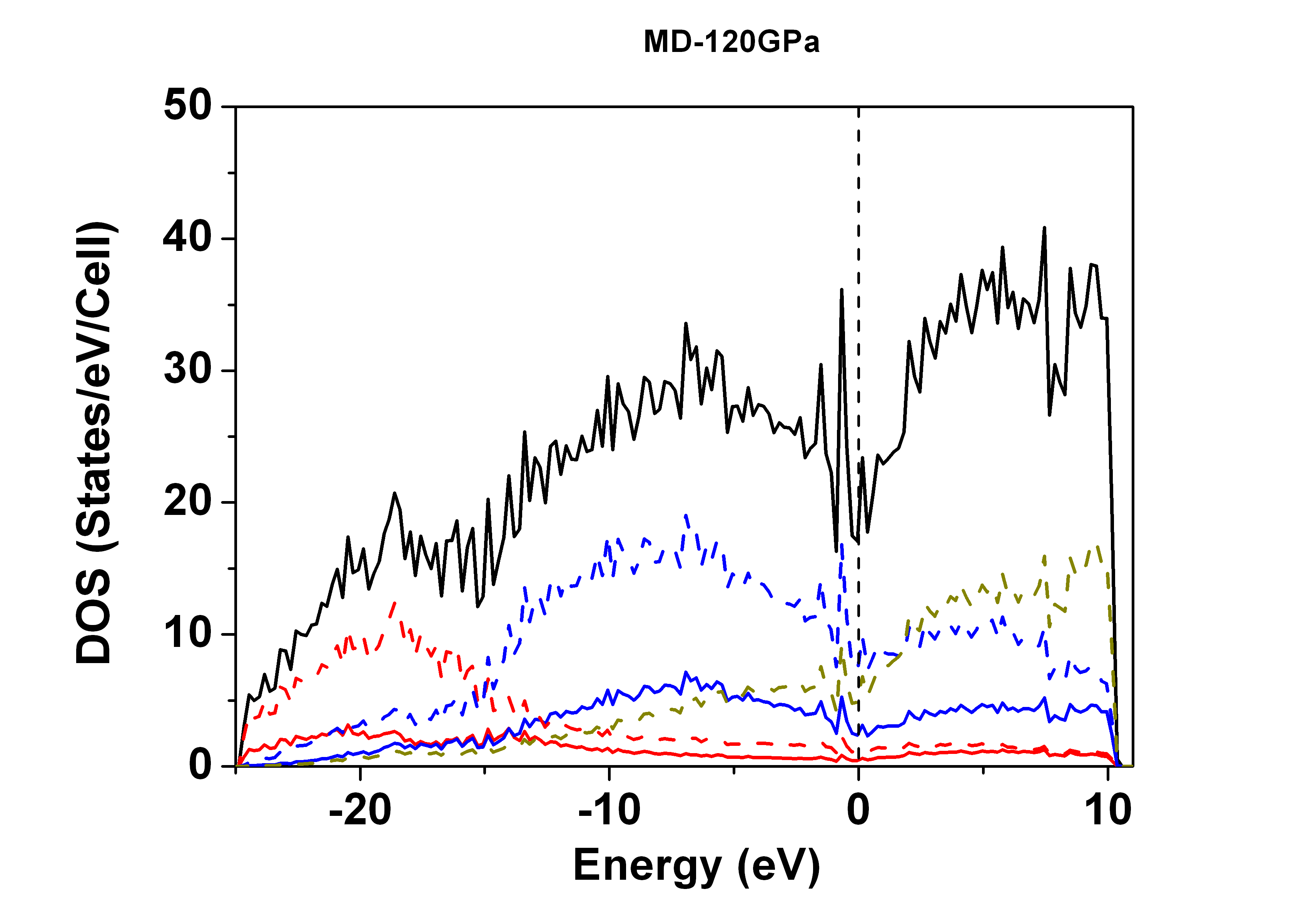


**Fig. S6** The calculated density of states of CS_2_ structure by MD simulation at 120 GPa.


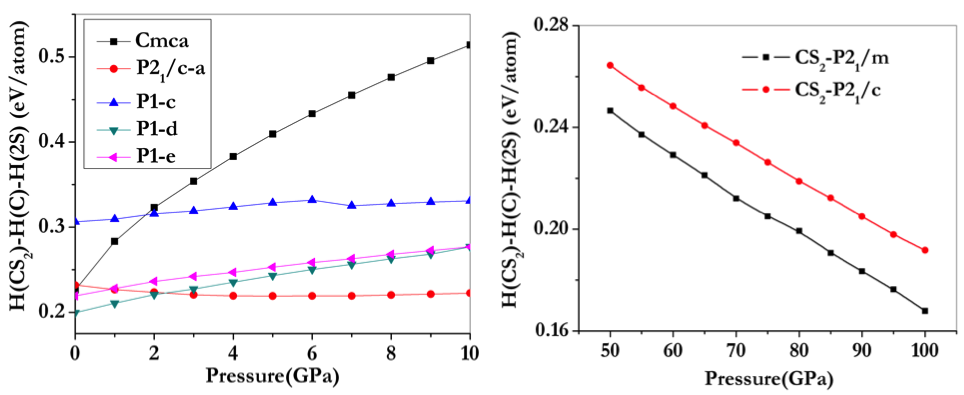


**Fig. S7** The calculated enthalpies of candidate structures for CS_2_ at high pressures.

**CS2-2GPa.cif**

1.

data_1-P

_audit_creation_date 2014-11-26

_audit_creation_method 'Materials Studio'

_symmetry_space_group_name_H-M 'P1'

_symmetry_Int_Tables_number 1

_symmetry_cell_setting triclinic

loop_

_symmetry_equiv_pos_as_xyz

x,y,z

_cell_length_a 5.1341

_cell_length_b 7.1345

_cell_length_c 6.8681

_cell_angle_alpha 68.5215

_cell_angle_beta 88.6769

_cell_angle_gamma 91.4605

loop_

_atom_site_label

_atom_site_type_symbol

_atom_site_fract_x

_atom_site_fract_y

_atom_site_fract_z

_atom_site_U_iso_or_equiv

_atom_site_adp_type

_atom_site_occupancy

C1 C 0.61820 0.67422 0.05054 0.01267 Uiso 1.00

C2 C 0.30188 0.38288 0.13926 0.01267 Uiso 1.00

C3 C 0.45448 0.40898 0.95944 0.01267 Uiso 1.00

C4 C 0.63911 0.57552 0.90882 0.01267 Uiso 1.00

S1 S 0.04902 0.20755 0.24763 0.01267 Uiso 1.00

S2 S 0.26989 0.94948 0.40778 0.01267 Uiso 1.00

S3 S 0.37905 0.26447 0.80835 0.01267 Uiso 1.00

S4 S 0.13439 0.83982 0.70469 0.01267 Uiso 1.00

S5 S 0.77991 0.88710 0.05984 0.01267 Uiso 1.00

S6 S 0.83579 0.63466 0.68226 0.01267 Uiso 1.00

S7 S 0.71282 0.12041 0.77837 0.01267 Uiso 1.00

S8 S 0.37864 0.56160 0.24171 0.01267 Uiso 1.00

2.

data_g8-740\(2)

_audit_creation_date 2014-04-09

_audit_creation_method 'Materials Studio'

_symmetry_space_group_name_H-M 'P1'

_symmetry_Int_Tables_number 1

_symmetry_cell_setting triclinic

loop_

_symmetry_equiv_pos_as_xyz

x,y,z

_cell_length_a 11.6061

_cell_length_b 7.1507

_cell_length_c 5.0040

_cell_angle_alpha 90.0000

_cell_angle_beta 84.8892

_cell_angle_gamma 90.0000

loop_

_atom_site_label

_atom_site_type_symbol

_atom_site_fract_x

_atom_site_fract_y

_atom_site_fract_z

_atom_site_U_iso_or_equiv

_atom_site_adp_type

_atom_site_occupancy

C1 C 0.85043 0.29930 0.23248 0.00000 Uiso 1.00

S2 S 0.15027 0.03739 0.01719 0.00000 Uiso 1.00

S3 S 0.58525 0.99410 0.23187 0.00000 Uiso 1.00

C4 C 0.14957 0.79930 0.01752 0.00000 Uiso 1.00

S5 S 0.84973 0.53739 0.23281 0.00000 Uiso 1.00

S6 S 0.41475 0.49410 0.01813 0.00000 Uiso 1.00

C7 C 0.14957 0.70070 0.26752 0.00000 Uiso 1.00

S8 S 0.84973 0.96261 0.48281 0.00000 Uiso 1.00

S9 S 0.41475 0.00590 0.26813 0.00000 Uiso 1.00

C10 C 0.85043 0.20070 0.48248 0.00000 Uiso 1.00

S11 S 0.15027 0.46261 0.26719 0.00000 Uiso 1.00

S12 S 0.58525 0.50590 0.48187 0.00000 Uiso 1.00

C13 C 0.85043 0.29930 0.73248 0.00000 Uiso 1.00

S14 S 0.15027 0.03739 0.51719 0.00000 Uiso 1.00

S15 S 0.58525 0.99410 0.73187 0.00000 Uiso 1.00

C16 C 0.14957 0.79930 0.51752 0.00000 Uiso 1.00

S17 S 0.84973 0.53739 0.73281 0.00000 Uiso 1.00

S18 S 0.41475 0.49410 0.51813 0.00000 Uiso 1.00

C19 C 0.14957 0.70070 0.76752 0.00000 Uiso 1.00

S20 S 0.84973 0.96261 0.98281 0.00000 Uiso 1.00

S21 S 0.41475 0.00590 0.76813 0.00000 Uiso 1.00

C22 C 0.85043 0.20070 0.98248 0.00000 Uiso 1.00

S23 S 0.15027 0.46261 0.76719 0.00000 Uiso 1.00

S24 S 0.58525 0.50590 0.98187 0.00000 Uiso 1.00

3.

data_3-P

_audit_creation_date 2014-11-26

_audit_creation_method 'Materials Studio'

_symmetry_space_group_name_H-M 'P1'

_symmetry_Int_Tables_number 1

_symmetry_cell_setting triclinic

loop_

_symmetry_equiv_pos_as_xyz

x,y,z

_cell_length_a 4.1233

_cell_length_b 7.4914

_cell_length_c 8.5991

_cell_angle_alpha 110.5492

_cell_angle_beta 105.7928

_cell_angle_gamma 96.9165

loop_

_atom_site_label

_atom_site_type_symbol

_atom_site_fract_x

_atom_site_fract_y

_atom_site_fract_z

_atom_site_U_iso_or_equiv

_atom_site_adp_type

_atom_site_occupancy

C1 C 0.32293 0.91352 0.55669 0.01267 Uiso 1.00

C2 C 0.44676 0.83998 0.69093 0.01267 Uiso 1.00

C3 C 0.05598 0.77963 0.40086 0.01267 Uiso 1.00

C4 C 0.46924 0.10260 0.56637 0.01267 Uiso 1.00

S1 S 0.41559 0.15100 0.38705 0.01267 Uiso 1.00

S2 S 0.73765 0.30125 0.75426 0.01267 Uiso 1.00

S3 S 0.86286 0.56238 0.39958 0.01267 Uiso 1.00

S4 S 0.92029 0.81130 0.20507 0.01267 Uiso 1.00

S5 S 0.48719 0.55801 0.06577 0.01267 Uiso 1.00

S6 S 0.79535 0.93649 0.87401 0.01267 Uiso 1.00

S7 S 0.21044 0.61170 0.64629 0.01267 Uiso 1.00

S8 S 0.66110 0.29788 0.98408 0.01267 Uiso 1.00

4.

data_4-P

_audit_creation_date 2014-11-26

_audit_creation_method 'Materials Studio'

_symmetry_space_group_name_H-M 'P1'

_symmetry_Int_Tables_number 1

_symmetry_cell_setting triclinic

loop_

_symmetry_equiv_pos_as_xyz

x,y,z

_cell_length_a 8.2532

_cell_length_b 8.6897

_cell_length_c 7.2365

_cell_angle_alpha 78.6076

_cell_angle_beta 81.7245

_cell_angle_gamma 113.6192

loop_

_atom_site_label

_atom_site_type_symbol

_atom_site_fract_x

_atom_site_fract_y

_atom_site_fract_z

_atom_site_U_iso_or_equiv

_atom_site_adp_type

_atom_site_occupancy

C1 C 0.72946 0.46939 0.64662 0.01267 Uiso 1.00

C2 C 0.77035 0.02876 0.35176 0.01267 Uiso 1.00

C3 C 0.28475 0.72454 0.57441 0.01267 Uiso 1.00

C4 C 0.21572 0.77734 0.42729 0.01267 Uiso 1.00

C5 C 0.27054 0.53061 0.35338 0.01267 Uiso 1.00

C6 C 0.22965 0.97124 0.64824 0.01267 Uiso 1.00

C7 C 0.71525 0.27546 0.42559 0.01267 Uiso 1.00

C8 C 0.78428 0.22266 0.57271 0.01267 Uiso 1.00

S1 S 0.29205 0.38737 0.23523 0.01267 Uiso 1.00

S2 S 0.20775 0.11446 0.76618 0.01267 Uiso 1.00

S3 S 0.32910 0.07975 0.97969 0.01267 Uiso 1.00

S4 S 0.17028 0.42259 0.02221 0.01267 Uiso 1.00

S5 S 0.70795 0.61264 0.76477 0.01267 Uiso 1.00

S6 S 0.79225 0.88554 0.23382 0.01267 Uiso 1.00

S7 S 0.67090 0.92025 0.02031 0.01267 Uiso 1.00

S8 S 0.82972 0.57741 0.97778 0.01267 Uiso 1.00

S9 S 0.67069 0.45329 0.42873 0.01267 Uiso 1.00

S10 S 0.82887 0.04487 0.56976 0.01267 Uiso 1.00

S11 S 0.31826 0.83646 0.75192 0.01267 Uiso 1.00

S12 S 0.18194 0.66543 0.24982 0.01267 Uiso 1.00

S13 S 0.32931 0.54671 0.57127 0.01267 Uiso 1.00

S14 S 0.17113 0.95513 0.43024 0.01267 Uiso 1.00

S15 S 0.68174 0.16354 0.24808 0.01267 Uiso 1.00

S16 S 0.81806 0.33457 0.75018 0.01267 Uiso 1.00

5.

data_5-P

_audit_creation_date 2014-11-26

_audit_creation_method 'Materials Studio'

_symmetry_space_group_name_H-M 'P1'

_symmetry_Int_Tables_number 1

_symmetry_cell_setting triclinic

loop_

_symmetry_equiv_pos_as_xyz

x,y,z

_cell_length_a 4.8194

_cell_length_b 5.8465

_cell_length_c 9.2745

_cell_angle_alpha 96.0689

_cell_angle_beta 111.6740

_cell_angle_gamma 97.7921

loop_

_atom_site_label

_atom_site_type_symbol

_atom_site_fract_x

_atom_site_fract_y

_atom_site_fract_z

_atom_site_U_iso_or_equiv

_atom_site_adp_type

_atom_site_occupancy

C1 C 0.87165 0.13700 0.22937 0.01267 Uiso 1.00

C2 C 0.68786 0.25710 0.11746 0.01267 Uiso 1.00

C3 C 0.94179 0.15182 0.39341 0.01267 Uiso 1.00

C4 C 0.88452 0.95771 0.96772 0.01267 Uiso 1.00

S1 S 0.02640 0.92237 0.15797 0.01267 Uiso 1.00

S2 S 0.16582 0.98586 0.50136 0.01267 Uiso 1.00

S3 S 0.97865 0.78946 0.83685 0.01267 Uiso 1.00

S4 S 0.53021 0.60143 0.67781 0.01267 Uiso 1.00

S5 S 0.50787 0.47292 0.14357 0.01267 Uiso 1.00

S6 S 0.65309 0.15948 0.92488 0.01267 Uiso 1.00

S7 S 0.81073 0.29763 0.51964 0.01267 Uiso 1.00

S8 S 0.54154 0.57267 0.46176 0.01267 Uiso 1.00

6.

data_CONTCAR_HIGH-3198

_audit_creation_date 2013-08-14

_audit_creation_method 'Materials Studio'

_symmetry_space_group_name_H-M 'CMCA'

_symmetry_Int_Tables_number 64

_symmetry_cell_setting orthorhombic

loop_

_symmetry_equiv_pos_as_xyz

x,y,z

-x,-y+1/2,z+1/2

-x,y+1/2,-z+1/2

x,-y,-z

-x,-y,-z

x,y+1/2,-z+1/2

x,-y+1/2,z+1/2

-x,y,z

x+1/2,y+1/2,z

-x+1/2,-y,z+1/2

-x+1/2,y,-z+1/2

x+1/2,-y+1/2,-z

-x+1/2,-y+1/2,-z

x+1/2,y,-z+1/2

x+1/2,-y,z+1/2

-x+1/2,y+1/2,z

_cell_length_a 6.0993

_cell_length_b 5.4258

_cell_length_c 8.6117

_cell_angle_alpha 90.0000

_cell_angle_beta 90.0000

_cell_angle_gamma 90.0000

loop_

_atom_site_label

_atom_site_type_symbol

_atom_site_fract_x

_atom_site_fract_y

_atom_site_fract_z

_atom_site_U_iso_or_equiv

_atom_site_adp_type

_atom_site_occupancy

C1 C 0.00000 0.50000 -0.00000 0.01267 Uiso 1.00

S1 S 1.00000 0.20448 -0.62646 0.01267 Uiso 1.00

7.

data_7-Pa3

_audit_creation_date 2014-11-26

_audit_creation_method 'Materials Studio'

_symmetry_space_group_name_H-M 'PA-3'

_symmetry_Int_Tables_number 205

_symmetry_cell_setting cubic

loop_

_symmetry_equiv_pos_as_xyz

x,y,z

-x+1/2,-y,z+1/2

-x,y+1/2,-z+1/2

x+1/2,-y+1/2,-z

z,x,y

z+1/2,-x+1/2,-y

-z+1/2,-x,y+1/2

-z,x+1/2,-y+1/2

y,z,x

-y,z+1/2,-x+1/2

y+1/2,-z+1/2,-x

-y+1/2,-z,x+1/2

-x,-y,-z

x+1/2,y,-z+1/2

x,-y+1/2,z+1/2

-x+1/2,y+1/2,z

-z,-x,-y

-z+1/2,x+1/2,y

z+1/2,x,-y+1/2

z,-x+1/2,y+1/2

-y,-z,-x

y,-z+1/2,x+1/2

-y+1/2,z+1/2,x

y+1/2,z,-x+1/2

_cell_length_a 6.6376

_cell_length_b 6.6376

_cell_length_c 6.6376

_cell_angle_alpha 90.0000

_cell_angle_beta 90.0000

_cell_angle_gamma 90.0000

loop_

_atom_site_label

_atom_site_type_symbol

_atom_site_fract_x

_atom_site_fract_y

_atom_site_fract_z

_atom_site_U_iso_or_equiv

_atom_site_adp_type

_atom_site_occupancy

C1 C 0.50000 0.50000 0.50000 1.00000 Uiso 1.00

S1 S 0.36491 0.36491 0.36491 1.00000 Uiso 1.00

8.

data_8-P

_audit_creation_date 2014-11-26

_audit_creation_method 'Materials Studio'

_symmetry_space_group_name_H-M 'P1'

_symmetry_Int_Tables_number 1

_symmetry_cell_setting triclinic

loop_

_symmetry_equiv_pos_as_xyz

x,y,z

_cell_length_a 4.0618

_cell_length_b 9.0669

_cell_length_c 8.1376

_cell_angle_alpha 82.0234

_cell_angle_beta 83.0369

_cell_angle_gamma 89.0533

loop_

_atom_site_label

_atom_site_type_symbol

_atom_site_fract_x

_atom_site_fract_y

_atom_site_fract_z

_atom_site_U_iso_or_equiv

_atom_site_adp_type

_atom_site_occupancy

C1 C 0.68302 0.74204 0.17774 0.01267 Uiso 1.00

C2 C 0.91934 0.45461 0.65162 0.01267 Uiso 1.00

C3 C 0.90110 0.19466 0.31833 0.01267 Uiso 1.00

C4 C 0.28484 0.90692 0.78537 0.01267 Uiso 1.00

S1 S 0.45943 0.85907 0.25889 0.01267 Uiso 1.00

S2 S 0.06393 0.03375 0.84902 0.01267 Uiso 1.00

S3 S 0.67782 0.39780 0.81029 0.01267 Uiso 1.00

S4 S 0.50770 0.78243 0.71868 0.01267 Uiso 1.00

S5 S 0.14538 0.24922 0.16026 0.01267 Uiso 1.00

S6 S 0.90532 0.62352 0.09810 0.01267 Uiso 1.00

S7 S 0.65997 0.13905 0.47622 0.01267 Uiso 1.00

S8 S 0.15686 0.51358 0.49338 0.01267 Uiso 1.00

9.

data_CIF

_audit_creation_date 2004-04-23

_audit_creation_method Materials Studio

_symmetry_space_group_name_H-M P1

_symmetry_Int_Tables_number 1

_symmetry_cell_setting triclinic

loop_

_symmetry_equiv_pos_as_xyz

x,y,z

_cell_length_a 3.8718

_cell_length_b 6.4602

_cell_length_c 13.1349

_cell_angle_alpha 80.6593

_cell_angle_beta 92.2300

_cell_angle_gamma 115.7126

loop_

_atom_site_label

_atom_site_type_symbol

_atom_site_fract_x

_atom_site_fract_y

_atom_site_fract_z

_atom_site_U_iso_or_equiv

_atom_site_adp_type

_atom_site_occupancy

C C 0.81576 0.95528 0.38211 0.00000 Uiso 1.00

C C 0.74073 0.81537 0.88085 0.00000 Uiso 1.00

C C 0.40007 0.34551 0.13255 0.00000 Uiso 1.00

C C 0.19102 0.26142 0.62961 0.00000 Uiso 1.00

S S 0.75391 0.05874 0.88183 0.00000 Uiso 1.00

S S 0.79844 0.70710 0.39885 0.00000 Uiso 1.00

S S 0.72678 0.57181 0.87978 0.00000 Uiso 1.00

S S 0.83217 0.20274 0.36712 0.00000 Uiso 1.00

S S 0.18737 0.02218 0.62387 0.00000 Uiso 1.00

S S 0.46541 0.58931 0.15387 0.00000 Uiso 1.00

S S 0.19587 0.50077 0.63567 0.00000 Uiso 1.00

S S 0.33581 0.10118 0.11221 0.00000 Uiso 1.00

10.

data_10-P

_audit_creation_date 2014-11-26

_audit_creation_method 'Materials Studio'

_symmetry_space_group_name_H-M 'P1'

_symmetry_Int_Tables_number 1

_symmetry_cell_setting triclinic

loop_

_symmetry_equiv_pos_as_xyz

x,y,z

_cell_length_a 3.6388

_cell_length_b 7.7861

_cell_length_c 10.7150

_cell_angle_alpha 72.3779

_cell_angle_beta 82.7235

_cell_angle_gamma 78.4267

loop_

_atom_site_label

_atom_site_type_symbol

_atom_site_fract_x

_atom_site_fract_y

_atom_site_fract_z

_atom_site_U_iso_or_equiv

_atom_site_adp_type

_atom_site_occupancy

C1 C 0.57462 0.74377 0.69998 0.01267 Uiso 1.00

C2 C 0.23649 0.69498 0.34045 0.01267 Uiso 1.00

C3 C 0.56316 0.42380 0.51333 0.01267 Uiso 1.00

C4 C 0.38839 0.56220 0.43168 0.01267 Uiso 1.00

S1 S 0.08298 0.38651 0.17429 0.01267 Uiso 1.00

S2 S 0.98469 0.19296 0.93369 0.01267 Uiso 1.00

S3 S 0.54864 0.25254 0.25730 0.01267 Uiso 1.00

S4 S 0.42521 0.62219 0.83380 0.01267 Uiso 1.00

S5 S 0.48373 0.05058 0.91427 0.01267 Uiso 1.00

S6 S 0.77111 0.25243 0.61194 0.01267 Uiso 1.00

S7 S 0.05931 0.85725 0.22824 0.01267 Uiso 1.00

S8 S 0.72374 0.86272 0.56774 0.01267 Uiso 1.00

**CS2-60GPa.cif**

**1.**

data_CONTCAR-p21m-60GPa

_audit_creation_date 2014-01-15

_audit_creation_method 'Materials Studio'

_symmetry_space_group_name_H-M 'P21/M'

_symmetry_Int_Tables_number 11

_symmetry_cell_setting monoclinic

loop_

_symmetry_equiv_pos_as_xyz

x,y,z

-x,y+1/2,-z

-x,-y,-z

x,-y+1/2,z

_cell_length_a 10.9097

_cell_length_b 2.3356

_cell_length_c 4.3562

_cell_angle_alpha 90.0000

_cell_angle_beta 84.3754

_cell_angle_gamma 90.0000

loop_

_atom_site_label

_atom_site_type_symbol

_atom_site_fract_x

_atom_site_fract_y

_atom_site_fract_z

_atom_site_U_iso_or_equiv

_atom_site_adp_type

_atom_site_occupancy

C1 C 0.47253 0.25000 0.43407 0.01267 Uiso 1.00

S2 S 0.30972 0.25000 1.04668 0.01267 Uiso 1.00

S4 S 0.88829 0.25000 0.14911 0.01267 Uiso 1.00

S5 S 0.30897 0.25000 0.54606 0.01267 Uiso 1.00

C2 C 0.52748 0.75000 0.92049 0.01267 Uiso 1.00

S1 S 0.10452 0.75000 0.34248 0.01267 Uiso 1.00

2.

data_CONTCAR-P21c-60

_audit_creation_method 'Materials Studio'

_audit_creation_date 2014-02-03

_audit_update_record 2014-02-03

_chemical_formula_sum 'C4 S8'

_chemical_formula_weight 304.572

_cell_length_a 11.0330

_cell_length_b 2.3456

_cell_length_c 4.3456

_cell_angle_alpha 90.000

_cell_angle_beta 79.888

_cell_angle_gamma 90.000

_cell_volume 110.7

_symmetry_int_tables_number 14

_symmetry_space_group_name_H-M 'P 1 21/c 1'

_symmetry_space_group_name_Hall '-P_2ybc'

loop_

_symmetry_equiv_pos_site_id

_symmetry_equiv_pos_as_xyz

1 x,y,z

2 -x,1/2+y,1/2-z

3 -x,-y,-z

4 x,1/2-y,1/2+z

loop_

_atom_type_symbol

_atom_type_oxidation_number

_atom_type_radius_bond

C ? 1.200

S ? 1.200

loop_

_atom_site_label

_atom_site_type_symbol

_atom_site_fract_x

_atom_site_fract_y

_atom_site_fract_z

_atom_site_occupancy

_atom_site_symmetry_multiplicity

_atom_site_Wyckoff_symbol

_atom_site_attached_hydrogens

_atom_site_calc_flag

_atom_site_thermal_displace_type

_atom_site_u_iso_or_equiv

C1 C 0.4783 0.0303 1.1739 1.000 4 e ? d Uiso 0.01267

S1 S 0.3143 0.0951 1.2403 1.000 4 e ? d Uiso 0.01267

S5 S 0.8908 0.2512 1.4235 1.000 4 e ? d Uiso 0.01267

3.

data_13-C2m

_audit_creation_date 2014-11-26

_audit_creation_method 'Materials Studio'

_symmetry_space_group_name_H-M 'C2/M'

_symmetry_Int_Tables_number 12

_symmetry_cell_setting monoclinic

loop_

_symmetry_equiv_pos_as_xyz

x,y,z

-x,y,-z

-x,-y,-z

x,-y,z

x+1/2,y+1/2,z

-x+1/2,y+1/2,-z

-x+1/2,-y+1/2,-z

x+1/2,-y+1/2,z

_cell_length_a 4.2153

_cell_length_b 2.5532

_cell_length_c 11.7881

_cell_angle_alpha 90.0000

_cell_angle_beta 118.5975

_cell_angle_gamma 90.0000

loop_

_atom_site_label

_atom_site_type_symbol

_atom_site_fract_x

_atom_site_fract_y

_atom_site_fract_z

_atom_site_U_iso_or_equiv

_atom_site_adp_type

_atom_site_occupancy

C1 C -0.80171 -0.50000 0.02270 0.01267 Uiso 1.00

S1 S 0.62677 -0.50000 0.59421 0.01267 Uiso 1.00

S5 S 1.57113 -0.50000 0.80669 0.01267 Uiso 1.00

4.

data_14-C2c-60GPa

_audit_creation_date 2014-08-19

_audit_creation_method 'Materials Studio'

_symmetry_space_group_name_H-M 'C2/C'

_symmetry_Int_Tables_number 15

_symmetry_cell_setting monoclinic

loop_

_symmetry_equiv_pos_as_xyz

x,y,z

-x,y,-z+1/2

-x,-y,-z

x,-y,z+1/2

x+1/2,y+1/2,z

-x+1/2,y+1/2,-z+1/2

-x+1/2,-y+1/2,-z

x+1/2,-y+1/2,z+1/2

_cell_length_a 4.2094

_cell_length_b 2.3841

_cell_length_c 22.5161

_cell_angle_alpha 90.0000

_cell_angle_beta 101.2250

_cell_angle_gamma 90.0000

loop_

_atom_site_label

_atom_site_type_symbol

_atom_site_fract_x

_atom_site_fract_y

_atom_site_fract_z

_atom_site_U_iso_or_equiv

_atom_site_adp_type

_atom_site_occupancy

C1 C -0.32301 -0.50017 0.51046 0.01267 Uiso 1.00

S1 S 1.42894 -0.47650 1.30253 0.01267 Uiso 1.00

S9 S 1.24189 -0.49835 0.90734 0.01267 Uiso 1.00

5.

data_15\-P1-60GPa

_audit_creation_date 2014-11-26

_audit_creation_method 'Materials Studio'

_symmetry_space_group_name_H-M 'P1'

_symmetry_Int_Tables_number 1

_symmetry_cell_setting triclinic

loop_

_symmetry_equiv_pos_as_xyz

x,y,z

_cell_length_a 2.4021

_cell_length_b 9.4315

_cell_length_c 10.1804

_cell_angle_alpha 94.7916

_cell_angle_beta 89.7189

_cell_angle_gamma 89.2381

loop_

_atom_site_label

_atom_site_type_symbol

_atom_site_fract_x

_atom_site_fract_y

_atom_site_fract_z

_atom_site_U_iso_or_equiv

_atom_site_adp_type

_atom_site_occupancy

C1 C 0.21656 0.97156 0.60945 0.01267 Uiso 1.00

C2 C 0.22975 0.75031 0.46295 0.01267 Uiso 1.00

C3 C 0.22232 0.86532 0.37305 0.01267 Uiso 1.00

C4 C 0.21215 0.10663 0.25689 0.01267 Uiso 1.00

C5 C 0.71718 0.95278 0.39743 0.01267 Uiso 1.00

C6 C 0.71700 0.01586 0.54008 0.01267 Uiso 1.00

C7 C 0.71044 0.07556 0.31851 0.01267 Uiso 1.00

C8 C 0.23274 0.81264 0.60265 0.01267 Uiso 1.00

S1 S 0.21417 0.26519 0.18505 0.01267 Uiso 1.00

S2 S 0.13106 0.04324 0.77892 0.01267 Uiso 1.00

S3 S 0.23288 0.77307 0.21595 0.01267 Uiso 1.00

S4 S 0.72973 0.64584 0.01002 0.01267 Uiso 1.00

S5 S 0.84750 0.25276 0.71177 0.01267 Uiso 1.00

S6 S 0.26368 0.84551 0.89628 0.01267 Uiso 1.00

S7 S 0.19895 0.95247 0.09475 0.01267 Uiso 1.00

S8 S 0.73660 0.62641 0.41771 0.01267 Uiso 1.00

S9 S 0.26035 0.35315 0.91593 0.01267 Uiso 1.00

S10 S 0.73407 0.19634 0.50450 0.01267 Uiso 1.00

S11 S 0.73408 0.14301 -0.01227 0.01267 Uiso 1.00

S12 S 0.23475 0.39054 0.36430 0.01267 Uiso 1.00

S13 S 0.27083 0.46466 0.60583 0.01267 Uiso 1.00

S14 S 0.25084 0.52917 0.80574 0.01267 Uiso 1.00

S15 S 0.73706 0.74271 0.69668 0.01267 Uiso 1.00

S16 S 0.72329 0.50328 0.14301 0.01267 Uiso 1.00

6.

data_CONTCAR_group124

_audit_creation_date 2014-08-19

_audit_creation_method 'Materials Studio'

_symmetry_space_group_name_H-M 'PBCN'

_symmetry_Int_Tables_number 60

_symmetry_cell_setting orthorhombic

loop_

_symmetry_equiv_pos_as_xyz

x,y,z

-x+1/2,-y+1/2,z+1/2

-x,y,-z+1/2

x+1/2,-y+1/2,-z

-x,-y,-z

x+1/2,y+1/2,-z+1/2

x,-y,z+1/2

-x+1/2,y+1/2,z

_cell_length_a 2.3433

_cell_length_b 6.7886

_cell_length_c 14.0889

_cell_angle_alpha 90.0000

_cell_angle_beta 90.0000

_cell_angle_gamma 90.0000

loop_

_atom_site_label

_atom_site_type_symbol

_atom_site_fract_x

_atom_site_fract_y

_atom_site_fract_z

_atom_site_U_iso_or_equiv

_atom_site_adp_type

_atom_site_occupancy

S1 S -1.04422 0.12965 -0.34268 0.01267 Uiso 1.00

S9 S -1.16748 0.87698 -0.44540 0.01267 Uiso 1.00

C1 C -1.50000 0.55987 0.25000 0.01267 Uiso 1.00

C5 C -1.50000 0.17938 0.25000 0.01267 Uiso 1.00

7.

data_CONTCAR_HIGH-3631

_audit_creation_date 2014-08-19

_audit_creation_method 'Materials Studio'

_symmetry_space_group_name_H-M 'PNMA'

_symmetry_Int_Tables_number 62

_symmetry_cell_setting orthorhombic

loop_

_symmetry_equiv_pos_as_xyz

x,y,z

-x+1/2,-y,z+1/2

-x,y+1/2,-z

x+1/2,-y+1/2,-z+1/2

-x,-y,-z

x+1/2,y,-z+1/2

x,-y+1/2,z

-x+1/2,y+1/2,z+1/2

_cell_length_a 11.6149

_cell_length_b 2.2616

_cell_length_c 8.7029

_cell_angle_alpha 90.0000

_cell_angle_beta 90.0000

_cell_angle_gamma 90.0000

loop_

_atom_site_label

_atom_site_type_symbol

_atom_site_fract_x

_atom_site_fract_y

_atom_site_fract_z

_atom_site_U_iso_or_equiv

_atom_site_adp_type

_atom_site_occupancy

C1 C 0.64528 0.75000 0.10833 0.01267 Uiso 1.00

S1 S 0.23638 0.75000 0.69230 0.01267 Uiso 1.00

C5 C 0.12806 0.25000 0.29997 0.01267 Uiso 1.00

S5 S 0.01426 0.25000 0.16193 0.01267 Uiso 1.00

S9 S 0.18365 0.25000 0.95309 0.01267 Uiso 1.00

S13 S 0.92075 0.25000 0.42696 0.01267 Uiso 1.00

8.

data_group1901-2100_407

_audit_creation_date 2014-08-19

_audit_creation_method 'Materials Studio'

_symmetry_space_group_name_H-M 'P212121'

_symmetry_Int_Tables_number 19

_symmetry_cell_setting orthorhombic

loop_

_symmetry_equiv_pos_as_xyz

x,y,z

-x+1/2,-y,z+1/2

-x,y+1/2,-z+1/2

x+1/2,-y+1/2,-z

_cell_length_a 7.1760

_cell_length_b 2.3628

_cell_length_c 13.8774

_cell_angle_alpha 90.0000

_cell_angle_beta 90.0000

_cell_angle_gamma 90.0000

loop_

_atom_site_label

_atom_site_type_symbol

_atom_site_fract_x

_atom_site_fract_y

_atom_site_fract_z

_atom_site_U_iso_or_equiv

_atom_site_adp_type

_atom_site_occupancy

C1 C 0.71305 0.25075 0.60916 0.00000 Uiso 1.00

C2 C 0.63426 0.75051 0.58224 0.00000 Uiso 1.00

S3 S 1.05691 0.25027 1.01372 0.00000 Uiso 1.00

S4 S 0.60362 0.74814 1.18359 0.00000 Uiso 1.00

S5 S 0.63328 0.76330 0.79130 0.00000 Uiso 1.00

S6 S 0.83434 0.77444 0.89171 0.00000 Uiso 1.00

9.

data_CONTCAR_HIGH-1981

_audit_creation_date 2014-08-19

_audit_creation_method 'Materials Studio'

_symmetry_space_group_name_H-M 'CMCA'

_symmetry_Int_Tables_number 64

_symmetry_cell_setting orthorhombic

loop_

_symmetry_equiv_pos_as_xyz

x,y,z

-x,-y+1/2,z+1/2

-x,y+1/2,-z+1/2

x,-y,-z

-x,-y,-z

x,y+1/2,-z+1/2

x,-y+1/2,z+1/2

-x,y,z

x+1/2,y+1/2,z

-x+1/2,-y,z+1/2

-x+1/2,y,-z+1/2

x+1/2,-y+1/2,-z

-x+1/2,-y+1/2,-z

x+1/2,y,-z+1/2

x+1/2,-y,z+1/2

-x+1/2,y+1/2,z

_cell_length_a 2.3877

_cell_length_b 10.1271

_cell_length_c 9.4186

_cell_angle_alpha 90.0000

_cell_angle_beta 90.0000

_cell_angle_gamma 90.0000

loop_

_atom_site_label

_atom_site_type_symbol

_atom_site_fract_x

_atom_site_fract_y

_atom_site_fract_z

_atom_site_U_iso_or_equiv

_atom_site_adp_type

_atom_site_occupancy

C1 C 0.50000 0.21325 0.29434 0.01267 Uiso 1.00

S1 S 0.50000 0.04596 0.36376 0.01267 Uiso 1.00

S9 S 0.50000 0.16526 0.92514 0.01267 Uiso 1.00

10.

data_20-P1

_audit_creation_date 2014-11-26

_audit_creation_method 'Materials Studio'

_symmetry_space_group_name_H-M 'P1'

_symmetry_Int_Tables_number 1

_symmetry_cell_setting triclinic

loop_

_symmetry_equiv_pos_as_xyz

x,y,z

_cell_length_a 2.3709

_cell_length_b 8.9198

_cell_length_c 11.4966

_cell_angle_alpha 107.0387

_cell_angle_beta 86.6905

_cell_angle_gamma 82.5799

loop_

_atom_site_label

_atom_site_type_symbol

_atom_site_fract_x

_atom_site_fract_y

_atom_site_fract_z

_atom_site_U_iso_or_equiv

_atom_site_adp_type

_atom_site_occupancy

C1 C 0.33543 0.35703 0.32533 0.01267 Uiso 1.00

C2 C 0.61975 0.81698 0.14840 0.01267 Uiso 1.00

C3 C 0.08157 0.90665 0.13836 0.01267 Uiso 1.00

C4 C 0.08999 0.63755 0.66136 0.01267 Uiso 1.00

C5 C 0.64048 0.54440 0.64609 0.01267 Uiso 1.00

C6 C 0.74165 0.42481 0.52134 0.01267 Uiso 1.00

C7 C 0.44833 0.19896 0.25324 0.01267 Uiso 1.00

C8 C 0.24981 0.44830 0.45299 0.01267 Uiso 1.00

S1 S 0.81056 0.69494 0.90099 0.01267 Uiso 1.00

S2 S 0.15367 0.35212 0.95404 0.01267 Uiso 1.00

S3 S 0.39499 0.11533 0.85298 0.01267 Uiso 1.00

S4 S 0.98460 0.81846 0.76671 0.01267 Uiso 1.00

S5 S 0.98415 0.06594 0.28729 0.01267 Uiso 1.00

S6 S 0.48212 0.23320 0.11037 0.01267 Uiso 1.00

S7 S 0.09531 0.97623 0.01148 0.01267 Uiso 1.00

S8 S 0.47952 0.95258 0.45237 0.01267 Uiso 1.00

S9 S 0.14406 0.65379 0.46217 0.01267 Uiso 1.00

S10 S 0.86689 0.22334 0.50728 0.01267 Uiso 1.00

S11 S 0.40428 0.92115 0.61951 0.01267 Uiso 1.00

S12 S 0.65646 0.19780 0.69098 0.01267 Uiso 1.00

S13 S 0.62538 0.75681 0.28159 0.01267 Uiso 1.00

S14 S 0.71860 0.61351 0.05857 0.01267 Uiso 1.00

S15 S 0.29827 0.47562 0.22897 0.01267 Uiso 1.00

S16 S 0.65125 0.44131 0.77242 0.01267 Uiso 1.00

**CS2-100GPa.cif**

1.

data_15

_audit_creation_date 2014-07-15

_audit_creation_method 'Materials Studio'

_symmetry_space_group_name_H-M 'P21/M'

_symmetry_Int_Tables_number 11

_symmetry_cell_setting monoclinic

loop_

_symmetry_equiv_pos_as_xyz

x,y,z

-x,y+1/2,-z

-x,-y,-z

x,-y+1/2,z

_cell_length_a 10.4263

_cell_length_b 2.2559

_cell_length_c 4.2329

_cell_angle_alpha 90.0000

_cell_angle_beta 84.2010

_cell_angle_gamma 90.0000

loop_

_atom_site_label

_atom_site_type_symbol

_atom_site_fract_x

_atom_site_fract_y

_atom_site_fract_z

_atom_site_U_iso_or_equiv

_atom_site_adp_type

_atom_site_occupancy

C1 C 0.47112 0.25000 0.43410 0.01267 Uiso 1.00

S2 S 0.30389 0.25000 1.04686 0.01267 Uiso 1.00

S4 S 0.89346 0.25000 0.15249 0.01267 Uiso 1.00

S5 S 0.30389 0.25000 0.55015 0.01267 Uiso 1.00

C2 C 0.52890 0.75000 0.91976 0.01267 Uiso 1.00

S1 S 0.10404 0.75000 0.34782 0.01267 Uiso 1.00

2.

data_14

_audit_creation_date 2014-07-15

_audit_creation_method 'Materials Studio'

_symmetry_space_group_name_H-M 'P21/C'

_symmetry_Int_Tables_number 14

_symmetry_cell_setting monoclinic

loop_

_symmetry_equiv_pos_as_xyz

x,y,z

-x,y+1/2,-z+1/2

-x,-y,-z

x,-y+1/2,z+1/2

_cell_length_a 10.4622

_cell_length_b 2.2621

_cell_length_c 4.2558

_cell_angle_alpha 90.0000

_cell_angle_beta 79.4100

_cell_angle_gamma 90.0000

loop_

_atom_site_label

_atom_site_type_symbol

_atom_site_fract_x

_atom_site_fract_y

_atom_site_fract_z

_atom_site_U_iso_or_equiv

_atom_site_adp_type

_atom_site_occupancy

C1 C 0.47710 0.03663 0.67345 0.01267 Uiso 1.00

S1 S 0.30772 0.11631 0.74137 0.01267 Uiso 1.00

S5 S 0.89399 0.25353 0.92242 0.01267 Uiso 1.00

3.

data_CIF

_audit_creation_date 2004-04-23

_audit_creation_method Materials Studio

_symmetry_space_group_name_H-M P1

_symmetry_Int_Tables_number 1

_symmetry_cell_setting triclinic

loop_

_symmetry_equiv_pos_as_xyz

x,y,z

_cell_length_a 2.2810

_cell_length_b 5.1431

_cell_length_c 17.5835

_cell_angle_alpha 81.7602

_cell_angle_beta 87.7135

_cell_angle_gamma 91.1906

loop_

_atom_site_label

_atom_site_type_symbol

_atom_site_fract_x

_atom_site_fract_y

_atom_site_fract_z

_atom_site_U_iso_or_equiv

_atom_site_adp_type

_atom_site_occupancy

C C 0.69505 0.34055 0.97985 0.00000 Uiso 1.00

C C 0.20135 0.67052 0.02424 0.00000 Uiso 1.00

C C 0.26671 0.88369 0.25739 0.00000 Uiso 1.00

C C 0.22603 0.71409 0.93769 0.00000 Uiso 1.00

C C 0.75231 0.80825 0.30473 0.00000 Uiso 1.00

C C 0.18671 0.17494 0.97977 0.00000 Uiso 1.00

C C 0.68966 0.50143 0.04287 0.00000 Uiso 1.00

C C 0.72619 0.56726 0.91770 0.00000 Uiso 1.00

S S 0.36271 0.22086 0.47951 0.00000 Uiso 1.00

S S 0.36010 0.85035 0.78165 0.00000 Uiso 1.00

S S 0.34455 0.69214 0.18443 0.00000 Uiso 1.00

S S 0.73946 0.00466 0.37986 0.00000 Uiso 1.00

S S 0.91381 0.44297 0.58640 0.00000 Uiso 1.00

S S 0.31981 0.23817 0.73542 0.00000 Uiso 1.00

S S 0.53837 0.62313 0.44903 0.00000 Uiso 1.00

S S 0.21865 0.22407 0.25155 0.00000 Uiso 1.00

S S 0.22863 0.03356 0.89462 0.00000 Uiso 1.00

S S 0.75648 0.44311 0.83331 0.00000 Uiso 1.00

S S 0.17733 0.95369 0.06656 0.00000 Uiso 1.00

S S 0.25629 0.64388 0.68836 0.00000 Uiso 1.00

S S 0.68059 0.31368 0.13052 0.00000 Uiso 1.00

S S 0.95901 0.04780 0.63337 0.00000 Uiso 1.00

S S 0.70781 0.48150 0.34088 0.00000 Uiso 1.00

S S 0.84215 0.84631 0.54355 0.00000 Uiso 1.00

4.

data_P1

_audit_creation_date 2013-8-11

_audit_creation_method 'CALYPSO -> cif'

_symmetry_space_group_name_H-M 'P1'

_symmetry_Int_Tables_number 1

_symmetry_cell_setting triclinic

loop_

_symmetry_equiv_pos_as_xyz

x,y,z

_cell_length_a 14.1103

_cell_length_b 6.2134

_cell_length_c 2.3107

_cell_angle_alpha 87.7558

_cell_angle_beta 92.2372

_cell_angle_gamma 85.8553

loop_

_atom_site_label

_atom_site_type_symbol

_atom_site_fract_x

_atom_site_fract_y

_atom_site_fract_z

_atom_site_U_iso_or_equiv

_atom_site_adp_type

_atom_site_occupancy

C1 C 0.98258 0.65401 0.50520 0.00 Uiso 1.00

C2 C 0.56028 0.49425 0.88163 0.00 Uiso 1.00

C3 C 0.08442 0.64098 0.52303 0.00 Uiso 1.00

C4 C 0.41561 0.50895 0.36475 0.00 Uiso 1.00

C5 C 0.13439 0.56306 0.04310 0.00 Uiso 1.00

C6 C 0.46513 0.42697 0.88269 0.00 Uiso 1.00

C7 C 0.93090 0.59476 0.00080 0.00 Uiso 1.00

C8 C 0.61060 0.41470 0.39866 0.00 Uiso 1.00

S1 S 0.68214 0.79301 0.99208 0.00 Uiso 1.00

S2 S 0.76529 0.11983 0.49394 0.00 Uiso 1.00

S3 S 0.31012 0.38113 0.28287 0.00 Uiso 1.00

S4 S 0.16982 0.29380 0.02095 0.00 Uiso 1.00

S5 S 0.28103 0.03708 0.69884 0.00 Uiso 1.00

S6 S 0.24225 0.69453 0.11405 0.00 Uiso 1.00

S7 S 0.73099 0.45759 0.39911 0.00 Uiso 1.00

S8 S 0.83229 0.78445 0.93988 0.00 Uiso 1.00

S9 S 0.54124 0.78039 0.86842 0.00 Uiso 1.00

S10 S 0.88417 0.34394 0.00969 0.00 Uiso 1.00

S11 S 0.38897 0.78560 0.37513 0.00 Uiso 1.00

S12 S 0.02721 0.25162 0.90390 0.00 Uiso 1.00

S13 S 0.45509 0.14137 0.95414 0.00 Uiso 1.00

S14 S 0.10960 0.92920 0.49261 0.00 Uiso 1.00

S15 S 0.61130 0.13137 0.40848 0.00 Uiso 1.00

S16 S 0.95899 0.95141 0.49251 0.00 Uiso 1.00

5.

data_CONTCAR71

_audit_creation_date 2013-07-22

_audit_creation_method 'Materials Studio'

_symmetry_space_group_name_H-M 'P21'

_symmetry_Int_Tables_number 4

_symmetry_cell_setting monoclinic

loop_

_symmetry_equiv_pos_as_xyz

x,y,z

-x,y+1/2,-z

_cell_length_a 2.2811

_cell_length_b 13.2305

_cell_length_c 6.6062

_cell_angle_alpha 90.0000

_cell_angle_beta 90.2031

_cell_angle_gamma 90.0000

loop_

_atom_site_label

_atom_site_type_symbol

_atom_site_fract_x

_atom_site_fract_y

_atom_site_fract_z

_atom_site_U_iso_or_equiv

_atom_site_adp_type

_atom_site_occupancy

C1 C 0.99778 0.49995 0.56141 0.01267 Uiso 1.00

C3 C 0.50040 0.00006 0.93859 0.01267 Uiso 1.00

C5 C 0.00089 0.50045 1.18520 0.01267 Uiso 1.00

C7 C 0.50110 0.99957 0.31481 0.01267 Uiso 1.00

S1 S 0.46131 0.20050 0.37833 0.01267 Uiso 1.00

S3 S 0.96675 0.29935 1.12178 0.01267 Uiso 1.00

S5 S 0.50890 0.40599 0.87391 0.01267 Uiso 1.00

S7 S 0.01033 0.09404 0.62606 0.01267 Uiso 1.00

S9 S 0.00615 0.90131 0.62660 0.01267 Uiso 1.00

S11 S 0.50618 0.59872 0.87353 0.01267 Uiso 1.00

S13 S 0.03676 0.80028 0.37568 0.01267 Uiso 1.00

S15 S 0.50952 0.69977 1.12473 0.01267 Uiso 1.00

6.

data_g23

_audit_creation_date 2014-08-18

_audit_creation_method 'Materials Studio'

_symmetry_space_group_name_H-M 'PNMA'

_symmetry_Int_Tables_number 62

_symmetry_cell_setting orthorhombic

loop_

_symmetry_equiv_pos_as_xyz

x,y,z

-x+1/2,-y,z+1/2

-x,y+1/2,-z

x+1/2,-y+1/2,-z+1/2

-x,-y,-z

x+1/2,y,-z+1/2

x,-y+1/2,z

-x+1/2,y+1/2,z+1/2

_cell_length_a 4.4489

_cell_length_b 12.5939

_cell_length_c 3.5468

_cell_angle_alpha 90.0000

_cell_angle_beta 90.0000

_cell_angle_gamma 90.0000

loop_

_atom_site_label

_atom_site_type_symbol

_atom_site_fract_x

_atom_site_fract_y

_atom_site_fract_z

_atom_site_U_iso_or_equiv

_atom_site_adp_type

_atom_site_occupancy

S1 S 0.55880 0.94946 -0.75530 0.00000 Uiso 1.00

S5 S 0.79018 0.14808 -0.76406 0.00000 Uiso 1.00

C1 C 0.64638 0.75000 -0.92781 0.00000 Uiso 1.00

C5 C 0.68410 0.25000 -0.09022 0.00000 Uiso 1.00

7.

data_CIF

_audit_creation_date 2004-04-23

_audit_creation_method Materials Studio

_symmetry_space_group_name_H-M P1

_symmetry_Int_Tables_number 1

_symmetry_cell_setting triclinic

loop_

_symmetry_equiv_pos_as_xyz

x,y,z

_cell_length_a 2.2835

_cell_length_b 5.2173

_cell_length_c 17.2845

_cell_angle_alpha 85.3478

_cell_angle_beta 95.3832

_cell_angle_gamma 88.1072

loop_

_atom_site_label

_atom_site_type_symbol

_atom_site_fract_x

_atom_site_fract_y

_atom_site_fract_z

_atom_site_U_iso_or_equiv

_atom_site_adp_type

_atom_site_occupancy

C C 0.73715 0.14463 0.70932 0.00000 Uiso 1.00

C C 0.42675 0.49031 0.33917 0.00000 Uiso 1.00

C C 0.24789 0.51149 0.75629 0.00000 Uiso 1.00

C C 0.38328 0.70499 0.98754 0.00000 Uiso 1.00

C C 0.92407 0.63974 0.03510 0.00000 Uiso 1.00

C C 0.25299 -0.01585 0.71939 0.00000 Uiso 1.00

C C 0.95687 0.51871 0.38765 0.00000 Uiso 1.00

C C 0.76210 0.34812 0.76357 0.00000 Uiso 1.00

S S 0.54369 0.97286 0.56161 0.00000 Uiso 1.00

S S 0.02707 0.26810 0.46097 0.00000 Uiso 1.00

S S 0.33367 0.49129 0.91716 0.00000 Uiso 1.00

S S 0.83268 0.49097 0.18087 0.00000 Uiso 1.00

S S 0.35676 0.62439 0.52298 0.00000 Uiso 1.00

S S 0.18742 0.71190 0.66864 0.00000 Uiso 1.00

S S 0.94859 0.32446 0.06947 0.00000 Uiso 1.00

S S 0.36199 0.03430 0.97368 0.00000 Uiso 1.00

S S 0.70204 0.33375 0.61744 0.00000 Uiso 1.00

S S 0.66781 0.10031 0.19700 0.00000 Uiso 1.00

S S 0.30129 0.77547 0.81411 0.00000 Uiso 1.00

S S 0.98314 0.83826 0.41329 0.00000 Uiso 1.00

S S 0.94341 0.85333 0.10876 0.00000 Uiso 1.00

S S 0.82008 0.16119 0.85192 0.00000 Uiso 1.00

S S 0.36507 0.75111 0.27067 0.00000 Uiso 1.00

S S 0.43627 0.17609 0.31766 0.00000 Uiso 1.00

8.

data_28-P1

_audit_creation_date 2014-11-26

_audit_creation_method 'Materials Studio'

_symmetry_space_group_name_H-M 'P1'

_symmetry_Int_Tables_number 1

_symmetry_cell_setting triclinic

loop_

_symmetry_equiv_pos_as_xyz

x,y,z

_cell_length_a 2.2841

_cell_length_b 5.2202

_cell_length_c 17.2556

_cell_angle_alpha 85.3646

_cell_angle_beta 95.3660

_cell_angle_gamma 88.1007

loop_

_atom_site_label

_atom_site_type_symbol

_atom_site_fract_x

_atom_site_fract_y

_atom_site_fract_z

_atom_site_U_iso_or_equiv

_atom_site_adp_type

_atom_site_occupancy

C1 C 0.73803 0.14473 0.70937 1.00000 Uiso 1.00

C2 C 0.42561 0.49047 0.33917 1.00000 Uiso 1.00

C3 C 0.24843 0.51156 0.75634 1.00000 Uiso 1.00

C4 C 0.38295 0.70503 0.98744 1.00000 Uiso 1.00

C5 C 0.92361 0.63971 0.03505 1.00000 Uiso 1.00

C6 C 0.25374 0.98433 0.71935 1.00000 Uiso 1.00

C7 C 0.95571 0.51914 0.38774 1.00000 Uiso 1.00

C8 C 0.76313 0.34823 0.76360 1.00000 Uiso 1.00

S9 S 0.54447 0.97258 0.56150 1.00000 Uiso 1.00

S10 S 0.02549 0.26833 0.46101 1.00000 Uiso 1.00

S11 S 0.33572 0.49090 0.91701 1.00000 Uiso 1.00

S12 S 0.83367 0.49079 0.18116 1.00000 Uiso 1.00

S13 S 0.35838 0.62421 0.52285 1.00000 Uiso 1.00

S14 S 0.18841 0.71186 0.66854 1.00000 Uiso 1.00

S15 S 0.94801 0.32434 0.06925 1.00000 Uiso 1.00

S16 S 0.36108 0.03421 0.97375 1.00000 Uiso 1.00

S17 S 0.70266 0.33354 0.61740 1.00000 Uiso 1.00

S18 S 0.66655 0.10059 0.19698 1.00000 Uiso 1.00

S19 S 0.30232 0.77568 0.81415 1.00000 Uiso 1.00

S20 S 0.98160 0.83860 0.41332 1.00000 Uiso 1.00

S21 S 0.94335 0.85274 0.10887 1.00000 Uiso 1.00

S22 S 0.82008 0.16075 0.85207 1.00000 Uiso 1.00

S23 S 0.36277 0.75144 0.27066 1.00000 Uiso 1.00

S24 S 0.43631 0.17620 0.31764 1.00000 Uiso 1.00

9.

data_CONTCAR339

_audit_creation_date 2013-07-22

_audit_creation_method 'Materials Studio'

_symmetry_space_group_name_H-M 'CMCM'

_symmetry_Int_Tables_number 63

_symmetry_cell_setting orthorhombic

loop_

_symmetry_equiv_pos_as_xyz

x,y,z

-x,-y,z+1/2

-x,y,-z+1/2

x,-y,-z

-x,-y,-z

x,y,-z+1/2

x,-y,z+1/2

-x,y,z

x+1/2,y+1/2,z

-x+1/2,-y+1/2,z+1/2

-x+1/2,y+1/2,-z+1/2

x+1/2,-y+1/2,-z

-x+1/2,-y+1/2,-z

x+1/2,y+1/2,-z+1/2

x+1/2,-y+1/2,z+1/2

-x+1/2,y+1/2,z

_cell_length_a 2.5774

_cell_length_b 16.9164

_cell_length_c 2.2861

_cell_angle_alpha 90.0000

_cell_angle_beta 90.0000

_cell_angle_gamma 90.0000

loop_

_atom_site_label

_atom_site_type_symbol

_atom_site_fract_x

_atom_site_fract_y

_atom_site_fract_z

_atom_site_U_iso_or_equiv

_atom_site_adp_type

_atom_site_occupancy

C1 C 2.00000 0.47593 -0.25000 0.01267 Uiso 1.00

S1 S 1.50000 0.80118 -0.75000 0.01267 Uiso 1.00

S9 S 2.00000 1.09472 -0.75000 0.01267 Uiso 1.00

10.

data_P1

_audit_creation_date 2013-7-26

_audit_creation_method 'CALYPSO -> cif'

_symmetry_space_group_name_H-M 'P1'

_symmetry_Int_Tables_number 1

_symmetry_cell_setting triclinic

loop_

_symmetry_equiv_pos_as_xyz

x,y,z

_cell_length_a 9.6746

_cell_length_b 2.4983

_cell_length_c 8.9982

_cell_angle_alpha 89.9270

_cell_angle_beta 79.3000

_cell_angle_gamma 75.0400

loop_

_atom_site_label

_atom_site_type_symbol

_atom_site_fract_x

_atom_site_fract_y

_atom_site_fract_z

_atom_site_U_iso_or_equiv

_atom_site_adp_type

_atom_site_occupancy

C1 C 0.02362 0.97840 0.43006 0.00 Uiso 1.00

C2 C 0.98570 0.01328 0.95251 0.00 Uiso 1.00

C3 C 0.52362 0.47835 0.43005 0.00 Uiso 1.00

C4 C 0.48570 0.51323 0.95251 0.00 Uiso 1.00

C5 C 0.97638 0.02165 0.79420 0.00 Uiso 1.00

C6 C 0.01430 0.98677 0.27175 0.00 Uiso 1.00

C7 C 0.47638 0.52160 0.79419 0.00 Uiso 1.00

C8 C 0.51430 0.48672 0.27174 0.00 Uiso 1.00

S1 S 0.32433 0.67496 0.27678 0.00 Uiso 1.00

S2 S 0.65688 0.34152 0.71627 0.00 Uiso 1.00

S3 S 0.82433 0.17502 0.27678 0.00 Uiso 1.00

S4 S 0.15689 0.84157 0.71628 0.00 Uiso 1.00

S5 S 0.84312 0.15848 0.50798 0.00 Uiso 1.00

S6 S 0.17567 0.82504 0.94748 0.00 Uiso 1.00

S7 S 0.34311 0.65843 0.50797 0.00 Uiso 1.00

S8 S 0.67567 0.32498 0.94747 0.00 Uiso 1.00

S9 S 0.11764 0.38385 0.49672 0.00 Uiso 1.00

S10 S 0.88575 0.61253 0.05412 0.00 Uiso 1.00

S11 S 0.61764 0.88381 0.49671 0.00 Uiso 1.00

S12 S 0.38576 0.11248 0.05412 0.00 Uiso 1.00

S13 S 0.11424 0.38752 0.17014 0.00 Uiso 1.00

S14 S 0.88236 0.61619 0.72754 0.00 Uiso 1.00

S15 S 0.61425 0.88748 0.17013 0.00 Uiso 1.00

S16 S 0.38236 0.11615 0.72754 0.00 Uiso 1.00
